# Chalcogen-Bond-Assisted Formation of the N→C Dative Bonds in the Complexes between Chalcogenadiazoles/Chalcogenatriazoles and Fullerene C_60_

**DOI:** 10.3390/molecules29112685

**Published:** 2024-06-06

**Authors:** Yu Zhang, Weizhou Wang

**Affiliations:** College of Chemistry and Chemical Engineering, and Henan Key Laboratory of Function-Oriented Porous Materials, Luoyang Normal University, Luoyang 471934, China; yzhpaper@yahoo.com

**Keywords:** N→C dative bond, chalcogen bond, chalcogenadiazole, fullerene C_60_

## Abstract

The existence of the N→C dative bonds in the complexes between N-containing molecules and fullerenes have been verified both theoretically and experimentally. However, finding stable N→C dative bonds is still a highly challenging task. In this work, we investigated computationally the N→C dative bonds in the complexes formed by fullerene C_60_ with 1,2,5-chalcogenadiazoles, 2,1,3-benzochalcogenadiazoles, and 1,2,4,5-chalcogenatriazoles, respectively. It was found that the N→C dative bonds are formed along with the formation of the N–Ch···C (Ch = S, Se, Te) chalcogen bonds. In the gas phase, from S-containing complexes through Se-containing complexes to Te-containing complexes, the intrinsic interaction energies become more and more negative, which indicates that the N–Ch···C chalcogen bonds can facilitate the formation of the N→C dative bonds. The intrinsic interaction energies are compensated by the large deformation energy of fullerene C_60_. The total interaction energies of Te-containing complexes are negative, while both total interaction energies of the S-containing complexes and Se-containing complexes are positive. This means that the N→C dative bonds in the Te-containing complexes are more easily observed in experiments in comparison with those in the S-containing complexes and Se-containing complexes. This study provides a new theoretical perspective on the experimental observation of the N→C dative bonds in complexes involving fullerenes. Further, the formation of stable N→C dative bonds in the complexes involving fullerenes can significantly change the properties of fullerenes, which will greatly simulate and expand the application range of fullerenes.

## 1. Introduction

The “dative bond” is not a new term. In 1999, the International Union of Pure and Applied Chemistry (IUPAC) defined the dative bond as “the coordination bond formed upon interaction between molecular species, one of which serves as a donor and the other as an acceptor of the electron pair to be shared in the complex formed” [1]. Unlike covalent bonds, dative bonds have more significant polarity, weaker strength, and longer bond lengths. The distinctive features and nature of dative bonds have been reviewed extensively [2,3,4,5]. In a relatively recent review article, Nandi and Kozuch detailed the historical development of dative bonds and provided an outlook on their future advancement [5]. Currently, controversies related to dative bonds have become increasingly rare.

The well-known dative bond is the central bond in ammonia borane (H_3_N→BH_3_). Like the case in H_3_N→BH_3_, the dative bond is always represented by an arrow from the electron donor to the electron acceptor. There are many different kinds of dative bonds [2,3,4,5]. Fullerenes are of considerable interest due to their unusual structures and peculiar properties [6]. Through combined experimental and theoretical studies, Hobza and colleagues have discovered the existence of N→C dative bonds in the complexes between piperidine and fullerene molecules such as C_60_ and C_70_ [7,8,9]. The charge transfer from piperidine to fullerenes significantly changes the properties of fullerenes. Subsequently, it was found by quantum chemical calculations that the N→C dative bonds also exist in the complexes between piperidine and cyclo[*n*]carbons [10,11]. Further, the P→C dative bonds were computationally found in the complexes involving molecular carbon materials [12,13]. The N→C/P→C dative bonds in these complexes are unstable due to the large deformation energies of molecular carbon materials. This greatly limits the experimental observation and further applications of N→C/P→C dative bonds. It is still a highly challenging task to find stable N→C/P→C dative bonds in the complexes involving important molecular carbon materials.

The N→C dative bond in the 1:1 complex between piperidine and fullerene C_60_ is not very stable, whereas it is stable in the 2:1 complex between piperidine and fullerene C_60_ [7]. This is mainly caused by two factors. First, the strong N–H···N hydrogen bond between two piperidine molecules further stabilizes the 2:1 complex. Second, the stability of the N→C dative bond is greatly enhanced by the σ-bond cooperativity with the N–H···N hydrogen bond [7,14]. Note that the σ-bond cooperativity is a very common phenomenon between noncovalent bonds of the same or different types [15]. As is well known, besides hydrogen bonds, there are many other types of noncovalent bonds [16,17,18,19,20]. By similar principles, other noncovalent bonds should also enhance the N→C dative bonds in the complexes involving important molecular carbon materials. In this study, we focused on the chalcogen bonds [21,22,23] and explored how and why they can enhance the N→C dative bonds. Fullerene C_60_ was selected as a model molecule for the ball-shaped molecular carbon materials. The small molecules 1,2,5-chalcogenadiazoles, 2,1,3-benzochalcogenadiazoles, and 1,2,4,5-chalcogenatriazoles contain both nitrogen atoms and chalcogen atoms. They are ideal model molecules for studying the chalcogen-bond-enhanced N→C dative bonds. This is also one of the reasons why we are studying the influence of chalcogen bonds on the N→C dative bonds first.

## 2. Results and Discussion

### 2.1. Complexes between Chalcogenadiazoles and Fullerene C_60_

**Figure 1:**
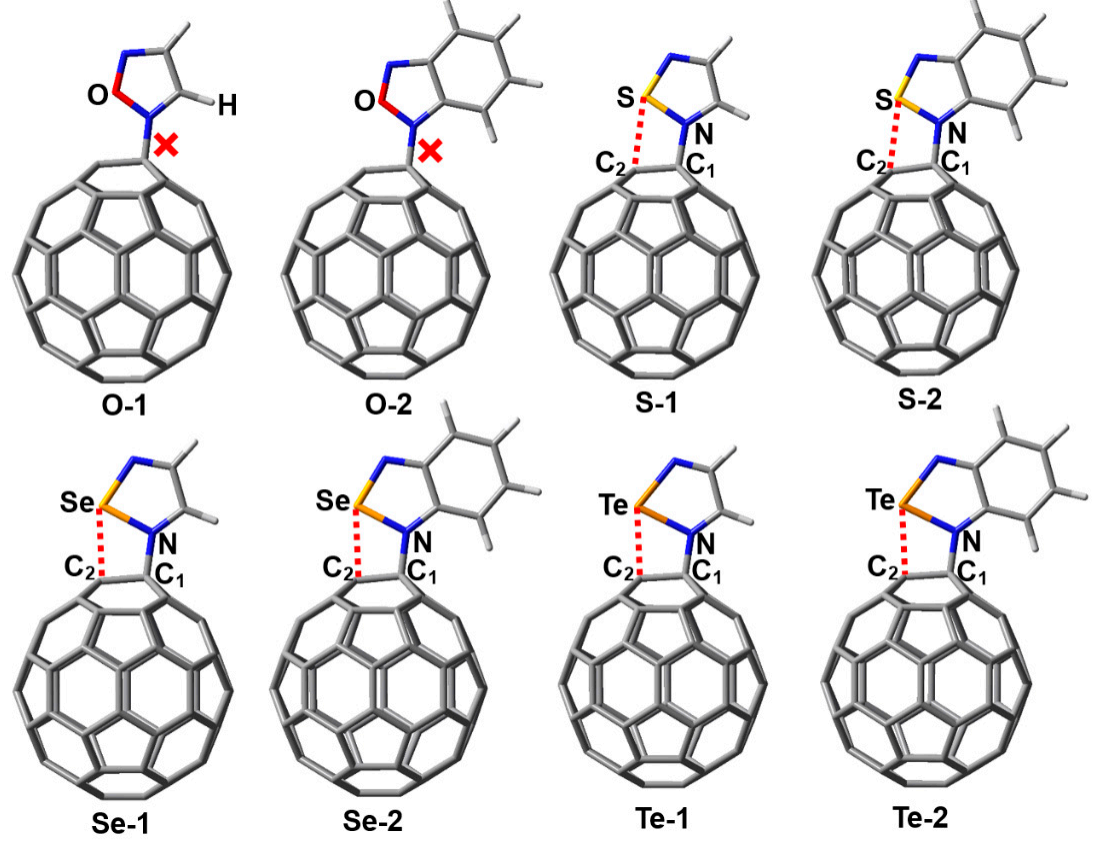
PBE0-D3/def2-TZVPP optimized structures of the complexes S-1, S-2, Se-1, Se-2, Te-1 and Te-2. The red dashed line indicates the possibility of a chalcogen bond.

At the outset of the research, we designed a series of complexes containing the N→C dative bonds (Figure 1). After the initial structures of the complexes O-1, O-2, S-1, S-2, Se-1, Se-2, Te-1, and Te-2 were fully optimized at the PBE0-D3/def2-TZVPP level of theory, we found that only the complexes O-1 and O-2 did not exist and instead transformed into corresponding complexes bound by the π···π stacking interactions. It can be clearly seen from Figure 1 that the fullerene C_60_ molecules undergo significant deformation in the PBE0-D3/def2-TZVPP optimized structures of the complexes S-1, S-2, Se-1, Se-2, Te-1, and Te-2. Table 1 lists the N···C_1_ and Ch···C_2_ interatomic distances, intrinsic interaction energies, total interaction energies, total dipole moments and Mulliken charges on N, C_1_, Ch and C_2_ for the complexes S-1, S-2, Se-1, Se-2, Te-1, and Te-2.

The Van der Waals radii of S, Se, Te and C are 1.80, 1.90, 2.06 and 1.70 Å, respectively [24]. The Ch···C_2_ interatomic distances in Table 1 are all smaller than the sum of the radii of two interacting atoms, which provides preliminary support for classifying the Ch···C_2_ interactions as chalcogen bonds. The N···C_1_ interatomic distances are in the range of 1.466–1.507 Å, which is clearly less than the interatomic distance of 1.606 Å for the N→C dative bond in the complex between piperidine and fullerene C_60_ [7]. This means that the covalent character of the N→C dative bonds in this study is significantly enhanced. In Table 1, the intrinsic interaction energies and total interaction energies contain the contributions of all the noncovalent interactions because it is difficult to completely separate N→C dative bonds from other noncovalent interactions. From the S-containing complexes through Se-containing complexes to Te-containing complexes, the intrinsic interaction energies become more and more negative. It is well known that the strength of a chalcogen bond increases in the order of S < Se < Te. Hence, such a result indicates that the N–Ch···C chalcogen bonds facilitate the formation of the N→C dative bonds. The intrinsic interaction energies are compensated by the large deformation energies of the monomers. Unlike the intrinsic interaction energies, the total interaction energies do not neglect the deformation energies of the monomers. As shown in Table 1, the total interaction energies of Te-containing complexes are negative, while both total interaction energies of the S-containing complexes and Se-containing complexes are positive. These results indicate that the N→C dative bonds in the Te-containing complexes are more easily observed in experiments in comparison with those in the S-containing complexes and Se-containing complexes.

At the PBE0-D3/def2-TZVPP theory level, the calculated total dipole moments of the monomers are in the range of 0–1.78 Debye. Compared to the monomers, the total dipole moments of the complexes significantly increase. This also conforms to the characteristics of dative bond formation. It is very convenient to employ the Mulliken charge for qualitative estimation of possible changes in electron distribution, although it is often criticized that Mulliken charge is arbitrary and heavily basis set dependent. Table 1 summarizes the Mulliken charges on N, C_1_, Ch and C_2_. The Mulliken charges on Ch atoms are all positive and the Mulliken charges on C_2_ atoms are all negative, which once again demonstrates the formation of chalcogen bonds.

In fact, we also calculated the complexes involving the 6:5 bonds of fullerene C_60_ at the PBE0-D3/def2-TZVPP theory level. Figure S1 in the Supplementary Materials illustrates the optimized structures and corresponding total interaction energies of the complexes S-2(6:5), Se-1(6:5), Se-2(6:5), Te-1(6:5) and Te-2(6:5). Unlike the results in Figure 1 and Table 1, the expected complex S-1(6:5) in Figure S1 does not exist, and the total interaction energies of the Te-containing complexes in Figure S1 are not negative. In line with our predictions based on the molecular electrostatic potentials of fullerene C_60_, the interactions between 6:5 bonds of fullerene C_60_ and N–Ch bonds of chalcogenazoles are much weaker than the interactions between 6:6 bonds of fullerene C_60_ and N–Ch bonds of chalcogenazoles. On the other hand, Table S1 in the Supplementary Materials also lists the calculated results for the complexes S-1, S-2, Se-1, Se-2, Te-1 and Te-2 at the low-cost PBE0-D3/6-31G(d) or PBE0-D3/SDD level of theory. Comparing the calculated results of Table 1 and Table S1, it can be observed that the low-cost PBE0-D3/6-31G(d) or PBE0-D3/SDD calculations yield qualitatively consistent results with those from high-cost PBE0-D3/def2-TZVPP calculations. Therefore, if the sole purpose is to conduct large-scale computational searches for stable N→C dative bonds in large complexes involving large molecular carbon materials, we recommend utilizing low-cost PBE0-D3/6-31G(d) or PBE0-D3/SDD calculations.

### 2.2. Molecular Electrostatic Potential Maps of Chalcogenadiazoles

**Figure 2:**
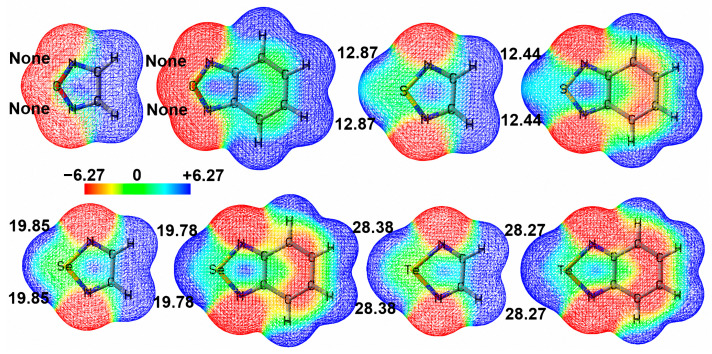
The electrostatic potential mapped electron density surfaces (isoval = 0.001 au) of 1,2,5-chalcogenadiazoles and 2,1,3-benzochalcogenadiazoles. The most positive electrostatic potential of each *σ*-hole (*V*_S,max_) is also shown. The electrostatic potentials are given in kcal/mol.

So far, there is still one question left unanswered: Why do the complexes O-1 and O-2 in Figure 1 not exist? To address this question, we plotted the electrostatic potential mapped electron density surfaces for 1,2,5-chalcogenadiazoles and 2,1,3-benzochalcogenadiazoles. Figure 2 shows that, except for 1,2,5-oxadiazole and 2,1,3-benzooxadiazole, other chalcogenadiazoles all have two *σ*-holes on the chalcogen atoms. In 1,2,5-oxadiazole and 2,1,3-benzooxadiazole, the electrostatic potentials on the extensions of the N–O bonds are all negative, which means that the N–O···C chalcogen bonds cannot be formed in the two complexes. When N–O···C chalcogen bonds are absent, N→C dative bonds are also absent; when N–Ch···C (Ch = S, Se, Te) chalcogen bonds are present, N→C dative bonds are also present. Clearly, the formation of chalcogen bonds determines whether N→C dative bonds form or not.

**Figure 3:**
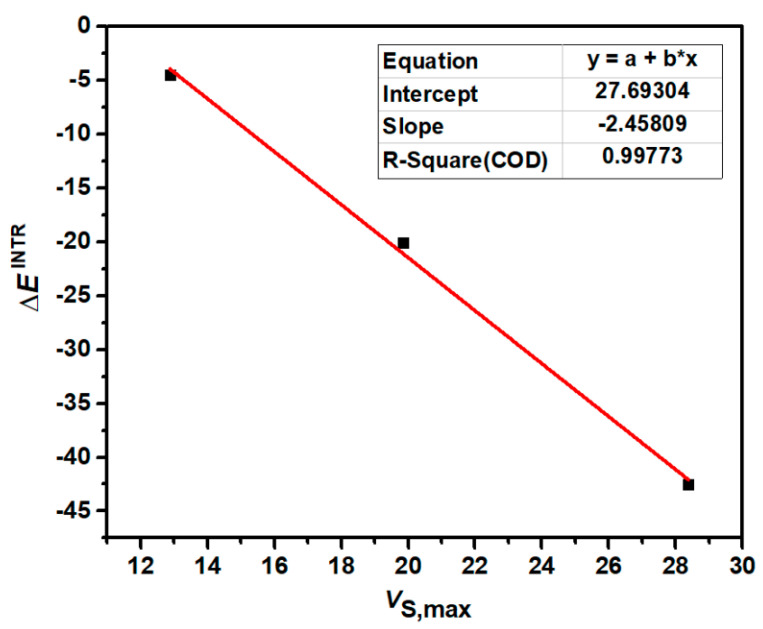
The correlation between *V*_S,max_ (kcal/mol) of 1,2,5-chalcogenadiazoles and ∆*E*^INTR^ (kcal/mol) of S-1, Se-1 and Te-1.

**Figure 4:**
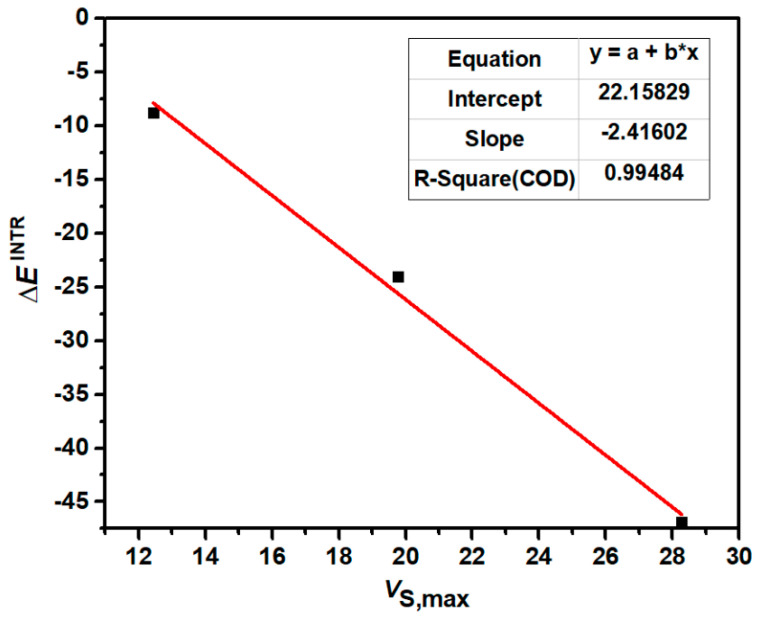
The correlation between *V*_S,max_ (kcal/mol) of 2,1,3-benzochalcogenadiazoles and ∆*E*^INTR^ (kcal/mol) of S-2, Se-2 and Te-2.

Figure 3 shows the correlation between the most positive electrostatic potentials of *σ*-holes of 1,2,5-chalcogenadiazoles and the intrinsic interaction energies of S-1, Se-1 and Te-1. Figure 4 shows the correlation between the most positive electrostatic potentials of *σ*-holes of 2,1,3-benzochalcogenadiazoles and the intrinsic interaction energies of S-2, Se-2 and Te-2. The correlation coefficients (R-Square) in Figure 3 and Figure 4 are both close to 1, indicating a very strong correlation between *V*_S,max_ and ∆*E*^INTR^. The value of *V*_S,max_ determines the strength of a chalcogen bond. At the same time, the more stable the complex, the more stable the N→C dative bond. Once again, it proves that the formation of chalcogen bonds determines the formation of N→C dative bonds.

### 2.3. AIM Analyses

As can be seen in Figure 1, according to the bonding situation of the nitrogen atom, it can be determined that a N→C dative bond is formed between the nitrogen atom and the carbon atom. In previous studies, the dative bonds with significant covalent bond components were also referred to as “dative/covalent bonds”, “covalent/dative bond” or “covalent dative bonds” [8,10,11,25]. Therefore, we will no longer discuss the nature of N→C dative bonds in this section and instead focus on the nature of chalcogen bonds.

**Figure 5:**
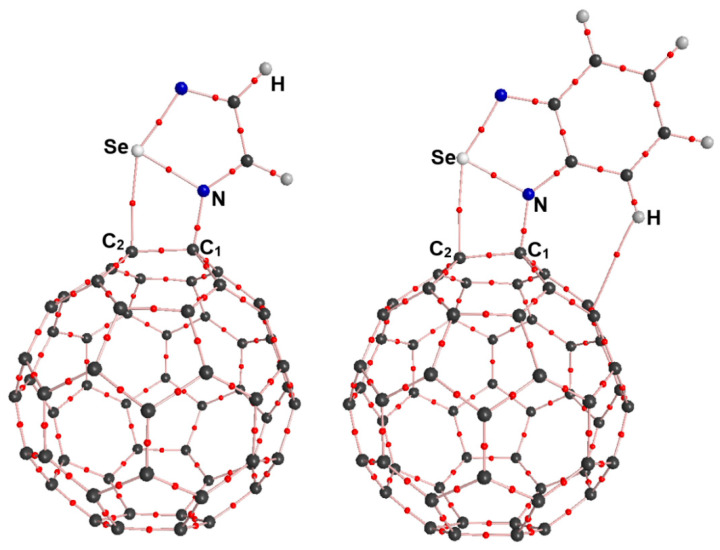
The bond critical points (small red dots) and bond paths of the complexes Se-1 and Se-2.

The AIM theory was often used to study the nature of the noncovalent bonds [26,27,28,29,30]. Figure 5 shows the bond critical points and bond paths for the complexes Se-1 and Se-2. The molecular graphs of S-1 and Te-1 are almost the same as the one of Se-1, and the molecular graphs of S-2 and Te-2 are almost the same as the one of Se-2 (Figure S2). For the sake of clarity, only the bond critical points and bond paths are shown in Figure 5 and Figure S2. The electron density, Laplacian of electron density, eigenvalues of the Hessian of electron density and ellipticity at the Ch···C_2_ bond critical point of each complex are summarized in Table 2.

The bond paths between Ch and C_2_ and the corresponding bond critical points can be clearly seen in the representative Figure 5. This is a necessary condition for the formation of the N–Ch···C_2_ chalcogen bond. The electron densities at the bond critical points of the N–Ch···C_2_ chalcogen bonds range from 0.0393 to 0.0651 au. In contrast, the electron densities at the bond critical points of hydrogen bonds range from 0.002 to 0.035 au [26,27]. The larger electron density means a much stronger noncovalent bond. Here, the N–Ch···C_2_ chalcogen bonds should belong to strong chalcogen bonds. The Laplacian of electron density is the sum of *λ*_1_, *λ*_2_ and *λ*_3_. According to AIM theory, the Laplacian of electron density is positive at the bond critical point of a noncovalent bond [31]. The values of ▽^2^*ρ*_b_ in Table 2 are all positive at the Ch···C_2_ bond critical point of each complex, which proves once again that the Ch···C_2_ contacts are of chalcogen bonds. The ellipticity ε can be calculated using the formula *λ*_1_/(*λ*_2_ − 1), and it can be used to assess the π character and structural stability of a bond. In Table 2, the values of *ε* are all very small, which indicates that the N–Ch···C_2_ chalcogen bonds are all very stable. This is consistent with the results of electron densities at the Ch···C_2_ bond critical points.

### 2.4. Complexes between Chalcogenatriazoles and Fullerene C_60_

It is meaningful to expand the complexes studied above to include more complexes. First, the fullerene C_60_ in the complexes can be replaced by other fullerene molecules or other molecular carbon materials. The results should be very similar. Second, the chalcogenadiazole can be replaced by its derivatives or other similar molecules. Here, we studied the complexes between 1,2,4,5-chalcogenatriazoles and fullerene C_60_.

**Figure 6:**
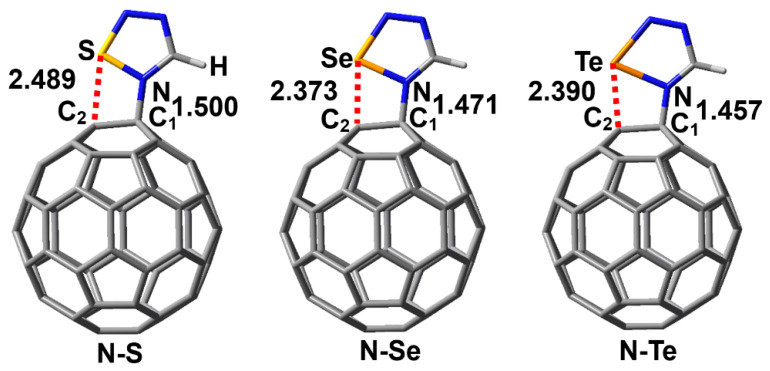
PBE0-D3/def2-TZVPP optimized structures of the complexes N–S, N–Se and N–Te. The red dashed lines represent the chalcogen bonds. The numbers shown are the interatomic distances (Å). The red dashed lines represent chalcogen bonds.

Figure 6 shows the PBE0-D3/def2-TZVPP optimized structures of the complexes N–S, N–Se and N–Te along with the N···C_1_ and Ch···C_2_ interatomic distances. Similarly, the Ch···C_2_ interatomic distances in Figure 6 are all smaller than the sum of the radii of two interacting atoms, which indicates the formation of the N–Ch···C_2_ chalcogen bonds. At the same time, the bonding situation in the three complexes clearly shows the formation of the N→C dative bonds. The intrinsic interaction energies of the complexes N–S, N–Se and N–Te are −0.11, −18.55 and −43.62 kcal/mol, respectively, whereas the total interaction energies of the complexes N–, N–Se and N–Te are +27.22, +17.44 and +1.21 kcal/mol, respectively. The positive total interaction energies indicate that these three complexes are difficult to form in the gas phase. In fact, the calculated total interaction energy of the complex between piperidine and fullerene C_60_ is also positive, but it has been detected by both FTIR spectra and NMR spectroscopy [7]. As pointed out above, the influence of the chemical environment around the complexes is also very important and must be considered during the experimental process. Aside from the 1,2,4,5-chalcogenatriazoles, we believe that there should be many similar nitrogen-containing organic compounds that can form stable chalcogen-bond-assisted N→C dative bonds with molecular carbon materials.

## 3. Materials and Methods

Unless otherwise stated, the structures and properties of all the monomers and complexes were calculated using the PBE0 density functional with Grimme’s D3 dispersion correction in conjunction with the def2-TZVPP basis set [32,33,34]. The Becke–Johnson damping function and ultrafine integration grid were used in all PBE0-D3/def2-TZVPP calculations [35]. The basis set superposition error was corrected with the conventional counterpoise method [36].

The calculations of noncovalent systems have always required extreme caution [37,38]. Bickelhaupt and colleagues carried out benchmark calculations for the chalcogen bonds with the type of D_2_Ch···A^–^ (Ch = S, Se; D, A = F, Cl) [38]. They found that some density functionals with specific dispersion corrections could give varying results for the chalcogen bonds. In this study, we calculated the complexes at the PBE0-D3/def2-TZVPP theory level. Our previous publications have shown that the PBE0-D3/def2-TZVPP calculations are reliable for the study of noncovalent systems [39,40]. Further, benchmark calculations on the dative bonds have been performed by employing a relatively small complex between fullerene C_20_ and piperidine [7]. The results again proved the reliability of the PBE0-D3/def2-TZVPP calculations for the study of the dative bonds.

It is also highly meaningful to study the reliability of results at low computational costs. Due to the generally large size of molecular carbon material systems, low-cost computations make studying these systems feasible. Using a small basis set 6-31G(d) for the S/Se-containing complexes and the small pseudopotential basis set SDD for the Te-containing complexes, the structures, harmonic frequencies, and energies of the complexes considered in this study were calculated at the PBE0-D3, PBE0, B3LYP-D3, B3LYP and M06-2X levels of theory, respectively. Let us add here that calculations involving heavy atom systems require the use of pseudopotential basis sets. The frequency calculations show that the structures of the complexes are true minima on their respective potential energy surfaces. For the geometries and energies of the complexes studied, quantitative differences exist between low-cost and high-cost computational results, but qualitative consistency can be achieved.

**Figure 7:**
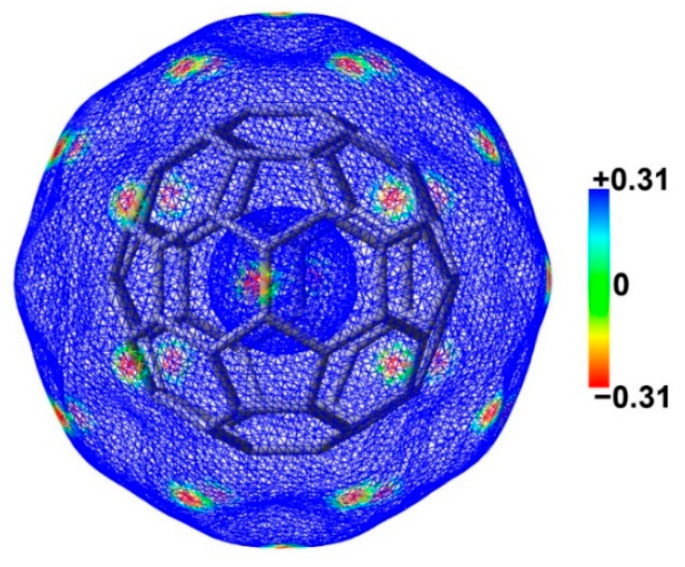
The electrostatic potential mapped electron density surface (isoval = 0.001 au) of fullerene C_60_. The color bar is in kcal/mol.

The fullerene C_60_ has two different types of carbon–carbon bonds as follows: the 6:6 bonds between two hexagons [1.401(10) Å] and the 6:5 bonds between a hexagon and a pentagon [1.458(6) Å] [41]. The 6:6 bonds are shorter than the 6:5 bonds and are more akin to the nature of double bonds. Figure 7 shows the electrostatic potential mapped electron density surface of fullerene C_60_. The electrostatic potentials above the 6:6 bonds are more negative than those above 6:5 bonds. The C atoms of fullerene C_60_ act as the electron donors upon the formation of the N–Ch···C chalcogen bonds. Therefore, only the interactions between 6:6 bonds of fullerene C_60_ and N–Ch bonds of chalcogenazoles were considered in this study.

The formation of the N–Ch···C chalcogen bonds was analyzed employing Bader’s “atoms in molecules” (AIM) theory [31]. Despite some controversies, the AIM theory remains a very useful tool for analyzing noncovalent interactions. The AIM analyses were performed with the AIM2000 software, version 2.0 [42]. The most positive electrostatic potential of each *σ*-hole of chalcogen atoms in 1,2,5-chalcogenadiazoles and 2,1,3-benzochalcogenadiazoles was calculated using the Multiwfn software, version 3.7 [43,44]. Other calculations were carried out with the GAUSSIAN 09 program package [45].

## 4. Conclusions

The N→C dative bonds in the complexes between chalcogenadiazoles and fullerene C_60_ have been investigated by employing quantum chemical calculations. The research scope has been further expanded to the complexes between 1,2,4,5-chalcogenatriazoles and fullerene C_60_. The results clearly show that the N–Ch···C chalcogen bonds facilitate the formation of the N→C dative bonds in these complexes. From then S-containing complexes through Se-containing complexes to Te-containing complexes, the complexes become increasingly stable as the N–Ch···C chalcogen bonds become stronger. Attractive N→C dative bonds and N–Ch···C chalcogen bonds together can overcome the significant repulsive deformation energy of fullerene C_60_. We therefore predict that the N→C dative bonds in the complexes between Te-containing molecules and fullerenes should be more easily observed experimentally and further applied. Indeed, following this line of thought, more stable complexes bound by the N→C dative bonds can be designed and synthesized. The formation of stable N→C dative bonds in the complexes containing fullerenes can significantly change the properties of fullerenes. Such a molecular modification strategy will greatly simulate and expand the application range of fullerenes.

In this study, we only focused on the chalcogen-bond-assisted N→C dative bonds. Naturally, the other noncovalent bonds such as halogen bonds, pnictogen bonds, tetrel bonds, etc., can also facilitate the N→C/P→C dative bonds in the complexes involving important molecular carbon materials. The relevant research is currently ongoing in our laboratory. On the other hand, all the calculations in this study were performed in the gas phase. It must be pointed out that the chalcogen-bond-assisted N→C dative bonds may be more stable in certain solvents. The solvation effect is also one of our future research focuses. We did not consider the case for the planar molecular carbon materials in this study. This issue also awaits further investigation in the future.

## Figures and Tables

**Table 1:** The N···C_1_ and Ch···C_2_ interatomic distances (*d*, Å), intrinsic interaction energies (∆*E*^INTR^, kcal/mol), total interaction energies (∆*E*, kcal/mol), total dipole moments (*µ*, Debye) and Mulliken charges (*q*, *e*) on N, C_1_, Ch and C_2_ of the complexes studied.

Complex	*d*(N···C_1_)	*d*(Ch···C_2_)	${∆E}^{INTR}$	∆	*µ*	*q*(N)	*q*(C_1_)	*q*(Ch)	*q*(C_2_)
S-1	1.504	2.569	−4.52	20.85	8.14	0.081	1.121	0.351	−0.507
S-2	1.500	2.557	−8.75	18.13	10.18	0.090	1.153	0.381	−0.545
Se-1	1.480	2.467	−20.05	11.40	5.60	0.007	0.923	0.341	−0.221
Se-2	1.478	2.468	−24.04	8.35	7.59	−0.004	0.952	0.383	−0.241
Te-1	1.467	2.471	−42.54	−4.11	3.39	−0.142	1.019	0.443	−0.245
Te-2	1.466	2.464	−46.88	−7.32	4.92	−0.150	1.043	0.497	−0.245

**Table 2:** The electron density (*ρ*_b_), Laplacian of electron density (▽^2^*ρ*_b_), eigenvalues of the Hessian of electron density (*λ*_1_, *λ*_2_, *λ*_3_) and ellipticity (*ε*) at the Ch···C_2_ bond critical point of each complex. All the values are given in atomic units.

Complex	*ρ*_b_	▽^2^*ρ*_b_	*λ*_1_	*λ*_2_	*λ*_3_	*ε*
S-1	0.0393	0.0659	−0.0336	−0.0257	0.1253	0.3091
S-2	0.0398	0.0683	−0.0345	−0.0259	0.1287	0.3305
Se-1	0.0550	0.0493	−0.0509	−0.0491	0.1493	0.0370
Se-2	0.0544	0.0517	−0.0495	−0.0489	0.1502	0.0136
Te-1	0.0645	0.0302	−0.0618	−0.0544	0.1464	0.1355
Te-2	0.0651	0.0313	−0.0623	−0.0559	0.1496	0.1138

## Data Availability

Data are contained within the article.

## Supplementary Materials

The following supporting information can be downloaded at: https://www.mdpi.com/article/10.3390/molecules29112685/s1, Figure S1: The PBE0-D3/def2-TZVPP optimized structures and corresponding total interaction energies of the complexes involving the 6:5 bonds of fullerene C_60_; Figure S2: The molecular graphs of the complexes S-1, S-2, Te-1 and Te-2; Table S1: The calculated results at the PBE0-D3/6-31G(d) or PBE0-D3/SDD level of theory; Cartesian atomic coordinates for the complexes and monomers optimized at the PBE0-D3/def2-TZVPP theory level; Cartesian atomic coordinates for the complexes optimized at a variety of low-cost levels of theory.
