# Supplementary material for: Chalcogen-Bond-Assisted Formation of the N→C Dative Bonds in the Complexes between Chalcogenadiazoles/Chalcogenatriazoles and Fullerene C60
Source: Molecules. 2024 Jun 6;29(11):2685. doi: 10.3390/molecules29112685 (PMC11173879; doi:10.3390/molecules29112685)
Supplement: Supplementary file 1 [file molecules-29-02685-s001.zip › molecules-3019891-supplementary.pdf]

Supplementary Materials

Chalcogen-Bond-Assisted Formation of the N→C Dative Bonds in the Complexes between Chalcogenadiazoles/Chalcogenatriazoles and Fullerene C_{60}

Yu Zhang and Weizhou Wang *

College of Chemistry and Chemical Engineering, and Henan Key Laboratory of Function-Oriented Porous Materials, Luoyang Normal University, Luoyang 471934, China

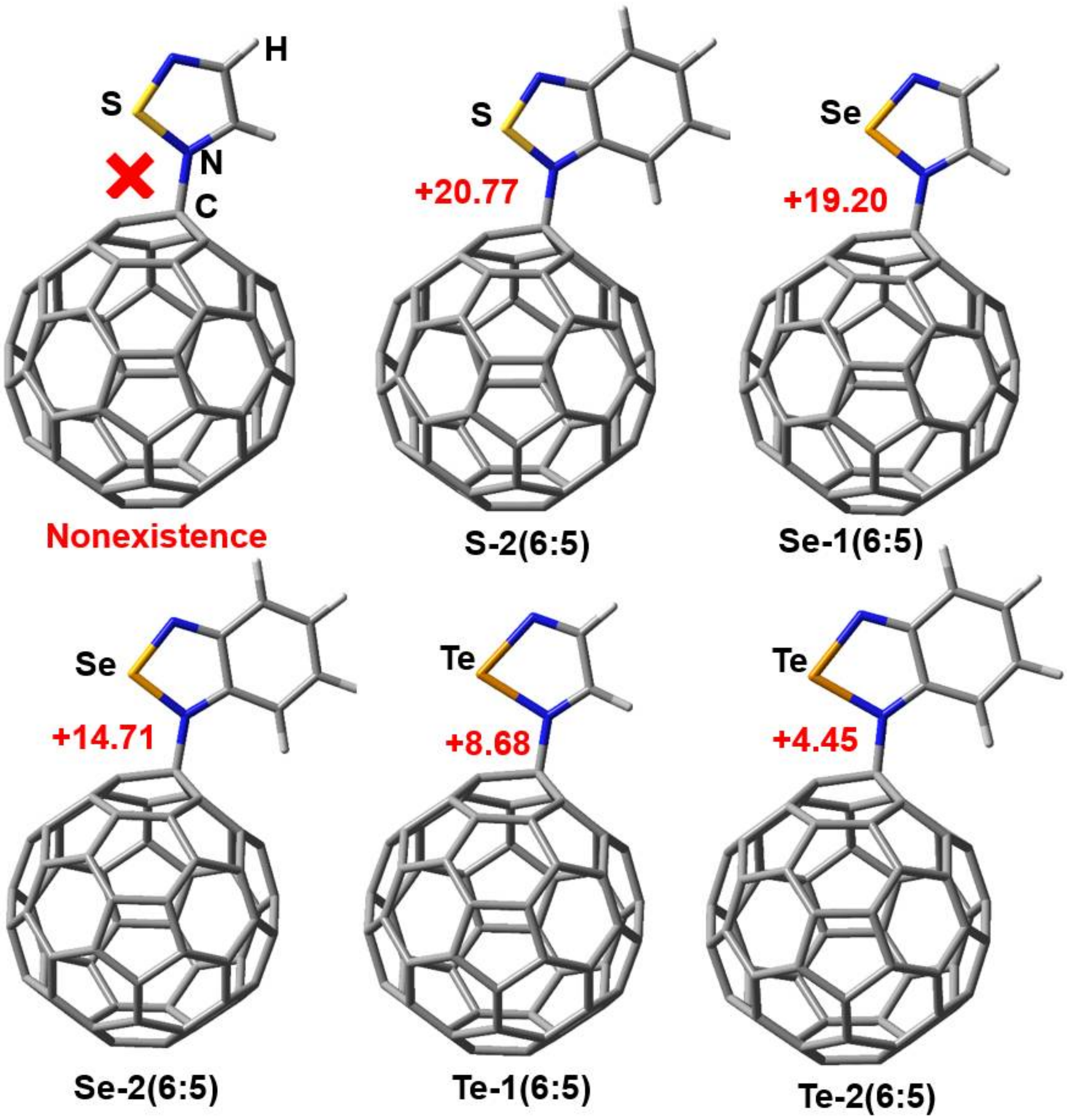

Figure S1. The PBE0-D3/def2-TZVPP optimized structures and corresponding

total interaction energies (red numbers, in kcal/mol) of the complexes involving the 6:5 bonds of fullerene C_{60}.

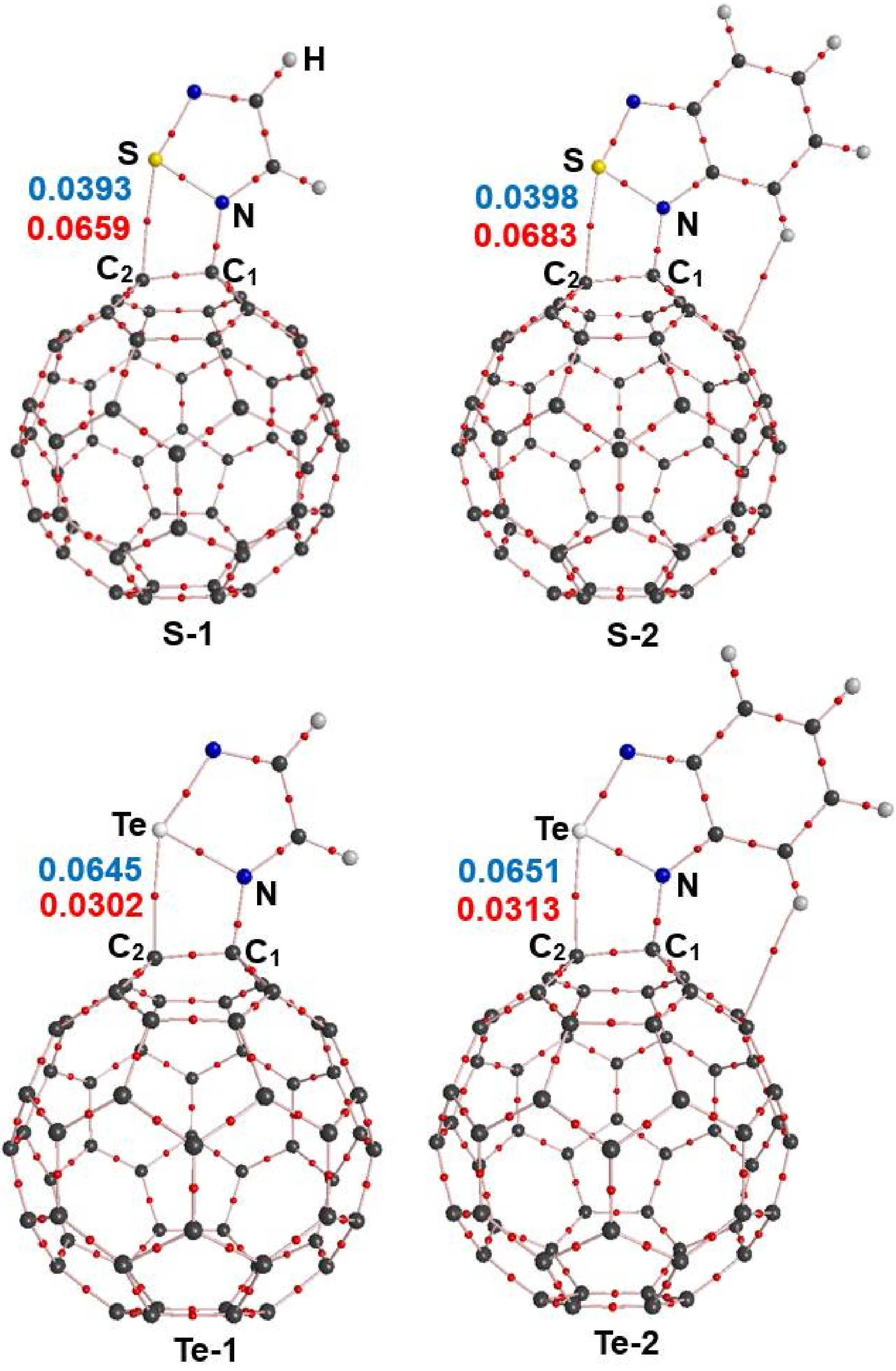

Figure S2. The molecular graphs of the complexes S-1, S-2, Te-1 and Te-2. The blue numbers are the values of electron densities (au) and the red numbers are the values of electron density Laplacians (au).

Table S1. The N···C_1 and Ch···C_2 interatomic distances (d, Å), intrinsic interaction energies (ΔE^{INTR}, kcal/mol), total interaction energies (ΔE, kcal/mol) and total dipole moments (μ, Debye) of the complexes studied. The S/Se-containing complexes were calculated at the PBE0-D3/6-31G(d) theory level and the Te-containing complexes were calculated at the PBE0-D3/SDD theory level.

Complex	d(N···C_1)	d(Ch···C_2)	ΔE^{INTR}	ΔE	μ
S-1	1.506	2.542	−3.18	19.15	7.60
S-2	1.501	2.537	−7.81	15.67	9.80
Se-1	1.478	2.360	−20.02	4.09	4.62
Se-2	1.475	2.377	−23.36	0.61	6.70
Te-1	1.479	2.430	−50.86	−20.56	3.24
Te-2	1.476	2.429	−55.63	−25.12	5.06

Cartesian coordinates of the complex S-1 optimized at the PBE0-D3/def2-TZVPP theory level:

C	0.008206	-0.080490	-3.439438
C	-0.837689	0.996245	-2.992254
C	-1.945969	0.420361	-2.270445
C	-0.581342	-1.327003	-2.997749

C	-1.800520	-1.031469	-2.299649
C	1.384797	0.079295	-3.450039
C	2.242253	-0.997538	-3.015942
C	3.346584	-0.417691	-2.289662
C	1.969090	1.326345	-3.005466
C	3.183007	1.019570	-2.289846
C	-0.277681	2.196001	-2.575319
C	1.155929	2.366076	-2.582918
C	1.519253	3.136585	-1.415040
C	-0.800684	2.860035	-1.414414
C	0.309005	3.444455	-0.690926
C	-2.473948	1.077793	-1.184548
C	-1.857827	2.284984	-0.722365
C	-1.857840	2.284816	0.722855
C	-3.066482	0.324422	0.000006
C	-2.473972	1.077518	1.184745
C	0.248705	-2.368322	-2.590004
C	-0.105247	-3.126466	-1.419036
C	1.105025	-3.438504	-0.693386
C	1.678951	-2.189894	-2.584852
C	2.212114	-2.857043	-1.416411
C	-2.120139	-1.737998	-1.150066

C	-2.754473	-1.148522	-0.000168
C	-2.120167	-1.738271	1.149605
C	-1.261334	-2.818572	-0.718001
C	-1.261349	-2.818740	0.717301
C	1.384730	0.078484	3.450070
C	1.969032	1.325639	3.005801
C	3.182963	1.019032	2.290132
C	2.242195	-0.998246	3.015737
C	3.346539	-0.418230	2.289614
C	0.008139	-0.081298	3.439405
C	-0.581401	-1.327707	2.997413
C	-1.800566	-1.032010	2.299358
C	-0.837746	0.995543	2.992460
C	-1.946013	0.419829	2.270496
C	1.155880	2.365470	2.583482
C	-0.277730	2.195397	2.575816
C	-0.800710	2.859704	1.415058
C	1.519226	3.136253	1.415792
C	0.308992	3.444293	0.691727
C	3.530558	1.757031	1.168668
C	2.682521	2.837622	0.722911
C	2.682535	2.837793	-0.722207

C	4.059397	1.092999	0.000160
C	3.530581	1.757306	-1.168201
C	1.678900	-2.190501	2.584357
C	2.212086	-2.857376	1.415769
C	1.105011	-3.438667	0.692586
C	0.248654	-2.368930	2.589438
C	-0.105275	-3.126799	1.418286
C	3.855358	-1.055255	1.169065
C	4.211285	-0.284927	0.000000
C	3.855381	-1.054980	-1.169253
C	3.273661	-2.299888	0.722815
C	3.273675	-2.299718	-0.723306
N	-4.569555	0.388140	-0.000007
N	-6.795704	-0.428612	0.000048
C	-5.422830	1.384503	-0.000059
C	-6.736874	0.883559	-0.000016
H	-7.636050	1.485746	0.000005
H	-5.081396	2.409319	-0.000085
S	-5.323262	-1.108448	0.000010

Cartesian coordinates of the complex S-2 optimized at the PBE0-D3/def2-TZVPP theory level:

C	-0.461570	-0.172085	3.439453
C	0.553833	0.746157	2.991823
C	1.550698	-0.008276	2.270850
C	-0.090354	-1.500220	2.998095
C	1.161855	-1.414458	2.301618
C	-1.791730	0.217562	3.450232
C	-2.818699	-0.699148	3.016458
C	-3.809277	0.058675	2.289871
C	-2.157567	1.545246	3.005883
C	-3.405885	1.447778	2.290190
C	0.203744	2.023360	2.576273
C	-1.180650	2.433061	2.583884
C	-1.408899	3.253824	1.415819
C	0.830333	2.588065	1.414375
C	-0.164295	3.352331	0.691372
C	2.184101	0.549607	1.184896
C	1.773926	1.841100	0.722672
C	1.773864	1.841134	-0.722753
C	2.649815	-0.292434	-0.000127
C	2.184005	0.549664	-1.185073
C	-1.084724	-2.386587	2.590174
C	-0.864162	-3.193376	1.418804

C	-2.109670	-3.296796	0.693056
C	-2.464165	-1.969193	2.584761
C	-3.102434	-2.536655	1.416128
C	1.354863	-2.162156	1.149159
C	2.072810	-1.684274	-0.000132
C	1.354768	-2.162106	-1.149386
C	0.326696	-3.084127	0.717315
C	0.326636	-3.084094	-0.717496
C	-1.792020	0.217718	-3.450085
C	-2.157820	1.545382	-3.005645
C	-3.406077	1.447882	-2.289851
C	-2.818952	-0.699011	-3.016266
C	-3.809470	0.058779	-2.289561
C	-0.461859	-0.171929	-3.439435
C	-0.090606	-1.500084	-2.998168
C	1.161663	-1.414354	-2.301794
C	0.553581	0.746293	-2.991849
C	1.550509	-0.008172	-2.270996
C	-1.180868	2.433179	-2.583688
C	0.203526	2.023477	-2.576213
C	0.830213	2.588130	-1.414342
C	-1.409019	3.253889	-1.415567

C	-0.164353	3.352362	-0.691220
C	-3.624280	2.233830	-1.168244
C	-2.605888	3.155646	-0.722407
C	-2.605827	3.155613	0.722756
C	-4.257673	1.668604	0.000210
C	-3.624181	2.233777	1.168637
C	-2.464382	-1.969076	-2.584656
C	-3.102553	-2.536591	-1.415996
C	-2.109728	-3.296765	-0.693042
C	-1.084941	-2.386470	-2.590205
C	-0.864281	-3.193311	-1.418890
C	-4.418396	-0.483358	-1.168941
C	-4.639158	0.335744	0.000196
C	-4.418298	-0.483411	1.169277
C	-4.055117	-1.808496	-0.722969
C	-4.055056	-1.808529	0.723214
N	4.134432	-0.505334	-0.000193
N	6.159448	-1.778655	-0.000317
C	7.647426	0.172278	-0.000213
H	8.548192	-0.426275	-0.000276
C	7.670816	1.534378	-0.000075
H	8.618960	2.056693	-0.000031

C	6.472613	2.298031	0.000031
H	6.547833	3.378378	0.000146
C	5.233813	1.722219	0.000008
H	4.328751	2.311502	0.000098
C	5.188357	0.316874	-0.000143
C	6.386276	-0.465908	-0.000251
S	4.593998	-2.109596	-0.000452

Cartesian coordinates of the complex Se-1 optimized at the PBE0-D3/def2-TZVPP theory level:

C	0.229651	-0.026419	-3.436477
C	-0.535051	1.111006	-2.990433
C	-1.682345	0.621343	-2.267796
C	-0.448355	-1.225819	-2.994258
C	-1.639163	-0.840183	-2.290055
C	1.613890	0.031087	-3.448824
C	2.389253	-1.106626	-3.013471
C	3.534316	-0.610430	-2.289366
C	2.289596	1.231375	-3.005944
C	3.476748	0.835222	-2.289763
C	0.113368	2.266522	-2.576142
C	1.555623	2.328563	-2.583361

C	1.975507	3.070351	-1.415818
C	-0.358115	2.967461	-1.415148
C	0.791445	3.467556	-0.691702
C	-2.157013	1.313833	-1.181673
C	-1.455436	2.473588	-0.722574
C	-1.455391	2.473674	0.722377
C	-2.817818	0.613577	0.000054
C	-2.156936	1.313972	1.181657
C	0.301333	-2.325927	-2.587333
C	-0.107510	-3.057357	-1.418729
C	1.076466	-3.457746	-0.692710
C	1.741525	-2.255603	-2.584305
C	2.223536	-2.960154	-1.416011
C	-2.015508	-1.526725	-1.151323
C	-2.638807	-0.899216	0.000142
C	-2.015438	-1.526587	1.151647
C	-1.237525	-2.660806	-0.718220
C	-1.237481	-2.660719	0.718633
C	1.614102	0.031504	3.448736
C	2.289782	1.231739	3.005669
C	3.476890	0.835499	2.289463
C	2.389438	-1.106262	3.013472

C	3.534457	-0.610153	2.289237
C	0.229863	-0.026004	3.436481
C	-0.448170	-1.225457	2.994448
C	-1.639021	-0.839907	2.290271
C	-0.534868	1.111367	2.990347
C	-1.682204	0.621616	2.267837
C	1.555782	2.328875	2.582999
C	0.113527	2.266832	2.575875
C	-0.358028	2.967631	1.414825
C	1.975594	3.070522	1.415339
C	0.791488	3.467640	0.691249
C	3.878692	1.545582	1.168246
C	3.113265	2.685929	0.722381
C	3.113221	2.685842	-0.722883
C	4.355936	0.843155	-0.000178
C	3.878620	1.545440	-1.168657
C	1.741684	-2.255291	2.584486
C	2.223624	-2.959983	1.416247
C	1.076508	-3.457662	0.693076
C	0.301493	-2.325614	2.587610
C	-0.107422	-3.057186	1.419120
C	3.994799	-1.283669	1.168920

C	4.407045	-0.541906	-0.000096
C	3.994727	-1.283810	-1.168996
C	3.323638	-2.482301	0.723110
C	3.323594	-2.482388	-0.723000
N	-4.290438	0.764112	0.000091
N	-6.657975	0.047348	0.000400
C	-5.106397	1.768968	0.000112
C	-6.464160	1.328386	0.000245
H	-7.299080	2.022738	0.000381
H	-4.751374	2.790945	0.000110
Se	-5.105349	-0.904717	0.000050

Cartesian coordinates of the complex Se-2 optimized at the PBE0-D3/def2-TZVPP theory level:

C	0.634982	-0.107579	-3.436778
C	-0.313921	0.881045	-2.990068
C	-1.361485	0.201438	-2.268446
C	0.172844	-1.405796	-2.995151
C	-1.067245	-1.230570	-2.292663
C	1.989024	0.186815	-3.449008
C	2.948532	-0.800814	-3.014119
C	3.991077	-0.115288	-2.289449

C	2.448589	1.485123	-3.005776
C	3.686218	1.298902	-2.289571
C	0.126761	2.130656	-2.576300
C	1.536923	2.439734	-2.583376
C	1.823093	3.242250	-1.415528
C	-0.457665	2.738239	-1.414323
C	0.588465	3.429159	-0.691155
C	-1.950322	0.800535	-1.181897
C	-1.452711	2.061049	-0.722442
C	-1.452707	2.060940	0.722760
C	-2.488687	-0.002653	0.000007
C	-1.950317	0.800358	1.182028
C	1.101000	-2.360731	-2.588138
C	0.824345	-3.151522	-1.419410
C	2.059390	-3.342760	-0.693212
C	2.507504	-2.043756	-2.584526
C	3.103710	-2.655155	-1.416283
C	-1.316933	-1.969427	-1.151027
C	-2.031471	-1.455330	-0.000107
C	-1.316931	-1.969603	1.150733
C	-0.356462	-2.954926	-0.718405
C	-0.356459	-2.955035	0.717956

C	1.989039	0.186291	3.449025
C	2.448603	1.484667	3.005988
C	3.686229	1.298555	2.289749
C	2.948545	-0.801271	3.013981
C	3.991087	-0.115635	2.289411
C	0.634997	-0.108100	3.436755
C	0.172857	-1.406250	2.994934
C	-1.067237	-1.230918	2.292479
C	-0.313907	0.880592	2.990200
C	-1.361476	0.201095	2.268482
C	1.536935	2.439342	2.583737
C	0.126773	2.130266	2.576620
C	-0.457658	2.738025	1.414738
C	1.823099	3.242036	1.416008
C	0.588468	3.429054	0.691670
C	3.960124	2.067307	1.168606
C	3.009924	3.059099	0.722869
C	3.009921	3.059209	-0.722421
C	4.551165	1.457749	0.000099
C	3.960118	2.067485	-1.168313
C	2.507515	-2.044148	2.584203
C	3.103716	-2.655370	1.415864

C	2.059393	-3.342865	0.692694
C	1.101011	-2.361124	2.587772
C	0.824351	-3.151737	1.418926
C	4.560500	-0.699740	1.168924
C	4.839257	0.101913	-0.000004
C	4.560495	-0.699563	-1.169053
C	4.105557	-1.995929	0.722906
C	4.105554	-1.995819	-0.723230
N	-3.961427	-0.127778	0.000004
Se	-4.458178	-1.906440	0.000027
N	-6.151483	-1.316447	-0.000065
C	-7.446717	0.735282	-0.000033
H	-8.381587	0.190947	-0.000064
C	-7.392533	2.091801	-0.000015
H	-8.307201	2.670881	-0.000029
C	-6.146025	2.781645	0.000014
H	-6.153599	3.864841	0.000024
C	-4.949353	2.130322	0.000022
H	-4.011449	2.666319	0.000037
C	-4.972957	0.718310	0.000009
C	-6.229467	-0.004274	-0.000020

Cartesian coordinates of the complex Te-1 optimized at the PBE0-D3/def2-TZVPP theory level:

C	-0.445313	0.009696	3.433866
C	0.262599	1.184518	2.988883
C	1.432605	0.754660	2.266154
C	0.289761	-1.154430	2.991155
C	1.457410	-0.709892	2.282571
C	-1.830333	-0.001891	3.447772
C	-2.548018	-1.177345	3.011703
C	-3.716916	-0.739152	2.289268
C	-2.565537	1.162958	3.006074
C	-3.730746	0.707806	2.289462
C	-0.443578	2.306628	2.576562
C	-1.886972	2.295357	2.583390
C	-2.343597	3.015324	1.416022
C	-0.008158	3.029684	1.415305
C	-1.180840	3.471386	0.691846
C	1.868893	1.466410	1.178791
C	1.112737	2.591623	0.722451
C	1.112655	2.591702	-0.722335
C	2.580456	0.810924	-0.000125
C	1.868765	1.466542	-1.178888

C	-0.403230	-2.290803	2.585295
C	0.041379	-3.001941	1.418883
C	-1.121218	-3.460802	0.692614
C	-1.845668	-2.294024	2.584089
C	-2.291749	-3.021534	1.416032
C	1.873244	-1.381966	1.153839
C	2.499632	-0.728846	-0.000201
C	1.873111	-1.381838	-1.154238
C	1.150437	-2.546610	0.718949
C	1.150354	-2.546530	-0.719391
C	-1.830725	-0.001517	-3.447600
C	-2.565879	1.163284	-3.005693
C	-3.731006	0.708055	-2.288997
C	-2.548360	-1.177019	-3.011576
C	-3.717176	-0.738904	-2.288962
C	-0.445704	0.010069	-3.433850
C	0.289421	-1.154105	-2.991349
C	1.457150	-0.709642	-2.282849
C	0.262259	1.184843	-2.988821
C	1.432351	0.754909	-2.266276
C	-1.887266	2.295638	-2.582963
C	-0.443872	2.306907	-2.576298

C	-0.008319	3.029838	-1.415013
C	-2.343758	3.015478	-1.415465
C	-1.180918	3.471461	-0.691372
C	-4.168150	1.397354	-1.168138
C	-3.460532	2.574403	-0.722376
C	-3.460450	2.574324	0.723013
C	-4.608882	0.671385	0.000280
C	-4.168018	1.397227	1.168727
C	-1.845962	-2.293744	-2.584163
C	-2.291910	-3.021381	-1.416134
C	-1.121297	-3.460727	-0.692897
C	-0.403524	-2.290521	-2.585532
C	0.041217	-3.001786	-1.419248
C	-4.143626	-1.434734	-1.168680
C	-4.592701	-0.714361	0.000204
C	-4.143494	-1.434861	1.168960
C	-3.414577	-2.598754	-0.722978
C	-3.414495	-2.598833	0.723049
N	4.028328	1.046947	-0.000214
N	6.531051	0.502161	0.000691
C	4.783828	2.086043	-0.000264
C	6.188637	1.740857	0.000069

H	6.937427	2.532079	0.000543
H	4.391941	3.096645	-0.000228
Te	4.969161	-0.798948	-0.000371

Cartesian coordinates of the complex Te-2 optimized at the PBE0-D3/def2-TZVPP theory level:

C	-0.804831	-0.060631	3.433980
C	0.089980	0.978672	2.988370
C	1.173313	0.359522	2.266726
C	-0.274860	-1.331592	2.992049
C	0.951337	-1.088344	2.284738
C	-2.172470	0.159721	3.447850
C	-3.076898	-0.878966	3.011715
C	-4.155961	-0.251238	2.289202
C	-2.702562	1.431127	3.006389
C	-3.927503	1.177507	2.289536
C	-0.419049	2.202991	2.577031
C	-1.843951	2.433771	2.583744
C	-2.173747	3.219836	1.416187
C	0.130832	2.841281	1.414984
C	-0.951018	3.474184	0.691944
C	1.725310	0.986944	1.179160

C	1.161121	2.219517	0.722688
C	1.161109	2.219688	-0.722171
C	2.325789	0.222653	0.000013
C	1.725292	0.987224	-1.178944
C	-1.148824	-2.335712	2.585409
C	-0.829916	-3.111196	1.418897
C	-2.052848	-3.369214	0.692442
C	-2.571333	-2.097251	2.583808
C	-3.133220	-2.739942	1.415835
C	1.245401	-1.818386	1.153378
C	1.966368	-1.276858	-0.000163
C	1.245384	-1.818113	-1.153823
C	0.339184	-2.847358	0.718651
C	0.339174	-2.847189	-0.719328
C	-2.172520	0.160540	-3.447776
C	-2.702606	1.431841	-3.006005
C	-3.927537	1.178051	-2.289194
C	-3.076943	-0.878251	-3.011875
C	-4.155995	-0.250694	-2.289196
C	-0.804882	-0.059816	-3.433978
C	-0.274903	-1.330881	-2.992357
C	0.951304	-1.087802	-2.285006

C	0.089936	0.979382	-2.988134
C	1.173279	0.360060	-2.266652
C	-1.843989	2.434384	-2.583133
C	-0.419087	2.203603	-2.576496
C	0.130810	2.841616	-1.414305
C	-2.173768	3.220172	-1.415384
C	-0.951029	3.474349	-0.691100
C	-4.243307	1.930608	-1.168245
C	-3.348756	2.972667	-0.722342
C	-3.348745	2.972496	0.723102
C	-4.799516	1.288755	0.000191
C	-4.243290	1.930331	1.168771
C	-2.571371	-2.096637	-2.584265
C	-3.133241	-2.739607	-1.416436
C	-2.052858	-3.369050	-0.693209
C	-1.148862	-2.335098	-2.585943
C	-0.829937	-3.110859	-1.419620
C	-4.693183	-0.865346	-1.168978
C	-5.015699	-0.080169	0.000030
C	-4.693166	-0.865623	1.168846
C	-4.169244	-2.134963	-0.723216
C	-4.169233	-2.135135	0.722774

N	3.791616	0.187028	0.000013
Te	4.377694	-1.781562	-0.000234
N	6.148381	-0.851095	-0.000490
C	7.218781	1.304764	-0.000044
H	8.192992	0.833792	-0.000277
C	7.065384	2.651399	0.000241
H	7.933376	3.298601	0.000256
C	5.767977	3.241618	0.000484
H	5.688764	4.322184	0.000683
C	4.629282	2.496340	0.000427
H	3.654933	2.962956	0.000575
C	4.745186	1.085252	0.000166
C	6.069435	0.451080	-0.000057

Cartesian coordinates of the complex N-S optimized at the PBE0-D3/def2-TZVPP theory level:

C	-0.002541	-0.073909	3.438686
C	0.833136	1.011563	2.991787
C	1.946326	0.447621	2.269170
C	0.597974	-1.314528	2.996818
C	1.812884	-1.006499	2.295688
C	-1.380342	0.072446	3.449552

C	-2.227143	-1.012850	3.014648
C	-3.337547	-0.443902	2.289431
C	-1.977026	1.313680	3.005491
C	-3.187663	0.994894	2.289769
C	0.260953	2.205636	2.575118
C	-1.174229	2.361395	2.582923
C	-1.545268	3.128625	1.415372
C	0.777176	2.875535	1.414471
C	-0.338147	3.448861	0.691286
C	2.467665	1.111051	1.184410
C	1.840566	2.312642	0.722425
C	1.840586	2.312614	-0.722465
C	3.064976	0.364100	0.000036
C	2.467696	1.111004	-1.184387
C	-0.221081	-2.363699	2.588675
C	0.140140	-3.119428	1.418745
C	-1.067126	-3.443057	0.692997
C	-1.653423	-2.200265	2.584437
C	-2.179836	-2.872561	1.416142
C	2.141253	-1.713149	1.151186
C	2.781486	-1.120761	0.000052
C	2.141278	-1.713185	-1.151077

C	1.293383	-2.799502	0.718132
C	1.293402	-2.799527	-0.718011
C	-1.380248	0.072325	-3.449605
C	-1.976944	1.313574	-3.005603
C	-3.187600	0.994814	-2.289903
C	-2.227061	-1.012956	-3.014685
C	-3.337485	-0.443982	-2.289519
C	-0.002447	-0.074029	-3.438695
C	0.598055	-1.314633	-2.996769
C	1.812944	-1.006579	-2.295615
C	0.833219	1.011457	-2.991810
C	1.946387	0.447539	-2.269140
C	-1.174157	2.361304	-2.583049
C	0.261025	2.205545	-2.575200
C	0.777216	2.875483	-1.414561
C	-1.545228	3.128575	-1.415536
C	-0.338128	3.448836	-0.691428
C	-3.542519	1.729270	-1.168520
C	-2.705327	2.818174	-0.722669
C	-2.705347	2.818200	0.722484
C	-4.064481	1.059588	-0.000080
C	-3.542551	1.729311	1.168350

C	-1.653354	-2.200356	-2.584417
C	-2.179798	-2.872611	-1.416113
C	-1.067108	-3.443082	-0.692917
C	-0.221011	-2.363790	-2.588610
C	0.140178	-3.119478	-1.418644
C	-3.840045	-1.086239	-1.169124
C	-4.203736	-0.319515	-0.000058
C	-3.840077	-1.086198	1.169045
C	-3.246686	-2.325264	-0.723048
C	-3.246706	-2.325238	0.723029
N	4.564364	0.417658	0.000064
N	6.752788	-0.386747	0.000006
C	5.464009	1.362184	-0.000024
H	5.219754	2.414251	-0.000147
N	6.734465	0.901240	0.000071
S	5.270747	-1.105109	0.000295

Cartesian coordinates of the complex N-Se optimized at the PBE0-D3/def2-TZVPP theory level:

C	0.221345	-0.017882	-3.434983
C	-0.531325	1.128372	-2.989372
C	-1.683480	0.652773	-2.266260

C	-0.468266	-1.209674	-2.992595
C	-1.652874	-0.810689	-2.284261
C	1.605815	0.024385	-3.448007
C	2.368378	-1.122045	-3.011852
C	3.519400	-0.638866	-2.289029
C	2.295011	1.216931	-3.005679
C	3.477291	0.807377	-2.289266
C	0.130398	2.276521	-2.575671
C	1.573222	2.322070	-2.582878
C	2.001432	3.059266	-1.415553
C	-0.332934	2.983006	-1.414778
C	0.821907	3.469892	-0.691462
C	-2.149457	1.350502	-1.180917
C	-1.436253	2.502928	-0.722289
C	-1.436273	2.502903	0.722340
C	-2.820314	0.659898	-0.000026
C	-2.149490	1.350461	1.180908
C	0.268269	-2.317751	-2.585518
C	-0.148692	-3.046797	-1.418800
C	1.031093	-3.460030	-0.692855
C	1.709497	-2.264889	-2.583992
C	2.183440	-2.974946	-1.416094

C	-2.040465	-1.498472	-1.153511
C	-2.675239	-0.867057	-0.000051
C	-2.040496	-1.498513	1.153405
C	-1.274689	-2.637170	-0.719182
C	-1.274709	-2.637195	0.719056
C	1.605720	0.024264	3.448055
C	2.294928	1.216825	3.005788
C	3.477228	0.807296	2.289393
C	2.368294	-1.122151	3.011881
C	3.519337	-0.638947	2.289107
C	0.221250	-0.018003	3.434991
C	-0.468348	-1.209779	2.992542
C	-1.652937	-0.810769	2.284189
C	-0.531407	1.128267	2.989400
C	-1.683542	0.652693	2.266239
C	1.573151	2.321979	2.583006
C	0.130327	2.276431	2.575758
C	-0.332973	2.982956	1.414877
C	2.001393	3.059216	1.415718
C	0.821888	3.469868	0.691609
C	3.887094	1.513047	1.168497
C	3.134545	2.661894	0.722737

C	3.134564	2.661919	-0.722554
C	4.355964	0.804976	0.000076
C	3.887127	1.513088	-1.168334
C	1.709426	-2.264980	2.583962
C	2.183401	-2.974996	1.416052
C	1.031073	-3.460055	0.692765
C	0.268198	-2.317842	2.585446
C	-0.148731	-3.046847	1.418692
C	3.972280	-1.317661	1.168855
C	4.393070	-0.580402	0.000052
C	3.972312	-1.317620	-1.168789
C	3.288711	-2.509050	0.722979
C	3.288731	-2.509025	-0.722974
N	-4.283131	0.811799	-0.000049
N	-6.606958	0.107850	-0.000060
C	-5.135179	1.765453	-0.000056
H	-4.888535	2.817917	-0.000034
Se	-5.047313	-0.916561	-0.000100
N	-6.460406	1.357645	-0.000075

Cartesian coordinates of the complex N-Te optimized at the PBE0-D3/def2-TZVPP theory level:

C	0.436253	0.018778	-3.432546
C	-0.257799	1.202432	-2.987853
C	-1.432604	0.787868	-2.264836
C	-0.311547	-1.136362	-2.989680
C	-1.472203	-0.677682	-2.277796
C	1.820752	-0.009583	-3.447120
C	2.523881	-1.193906	-3.010329
C	3.698404	-0.770228	-2.288915
C	2.570212	1.146077	-3.005812
C	3.729382	0.676537	-2.288954
C	0.462352	2.315785	-2.576085
C	1.905423	2.286550	-2.582925
C	2.370862	3.001018	-1.415714
C	0.035980	3.044353	-1.414908
C	1.213748	3.471484	-0.691577
C	-1.859172	1.504781	-1.178263
C	-1.090586	2.621255	-0.722177
C	-1.090604	2.621232	0.722250
C	-2.583408	0.861828	-0.000011
C	-1.859204	1.504744	1.178281
C	0.366713	-2.280780	-2.583585
C	-0.086774	-2.988162	-1.418805

C	1.070449	-3.461073	-0.692695
C	1.809196	-2.302951	-2.583759
C	2.246182	-3.036007	-1.416030
C	-1.900144	-1.349509	-1.156584
C	-2.536433	-0.689996	-0.000040
C	-1.900170	-1.349547	1.156495
C	-1.190883	-2.519081	-0.719862
C	-1.190900	-2.519105	0.719750
C	1.820668	-0.009703	3.447170
C	2.570138	1.145973	3.005921
C	3.729326	0.676458	2.289075
C	2.523808	-1.194010	3.010356
C	3.698348	-0.770307	2.288986
C	0.436169	0.018660	3.432563
C	-0.311620	-1.136464	2.989638
C	-1.472258	-0.677760	2.277741
C	-0.257872	1.202330	2.987896
C	-1.432660	0.787792	2.264838
C	1.905360	2.286462	2.583058
C	0.462289	2.315697	2.576184
C	0.035945	3.044305	1.415023
C	2.370827	3.000969	1.415883

C	1.213732	3.471460	0.691734
C	4.175042	1.360523	1.168503
C	3.481967	2.546165	0.722807
C	3.481984	2.546190	-0.722626
C	4.606434	0.628951	0.000071
C	4.175070	1.360563	-1.168347
C	1.809133	-2.303040	2.583730
C	2.246147	-3.036056	1.415985
C	1.070432	-3.461096	0.692607
C	0.366649	-2.280868	2.583520
C	-0.086809	-2.988210	1.418704
C	4.116414	-1.471356	1.168751
C	4.574575	-0.756425	0.000046
C	4.116444	-1.471316	-1.168696
C	3.373995	-2.626692	0.722947
C	3.374013	-2.626667	-0.722950
N	-4.017343	1.119594	-0.000029
N	-6.469824	0.583167	-0.000250
C	-4.799667	2.110296	-0.000027
H	-4.525972	3.157239	-0.000016
N	-6.184647	1.789406	-0.000120
Te	-4.923070	-0.816228	-0.000062

Cartesian coordinates of the monomer C_{60} optimized at the PBE0-D3/def2-TZVPP theory level:

C	1.168868	0.379788	3.306166
C	0.722400	-0.994299	3.306166
C	-0.722400	-0.994299	3.306166
C	0.000000	1.229021	3.306166
C	-1.168868	0.379788	3.306166
C	2.289659	0.743955	2.577832
C	2.289659	1.972976	1.818256
C	3.012060	1.738254	0.589235
C	3.012060	-0.250343	1.818256
C	3.458528	0.364167	0.589235
C	1.415087	-1.947701	2.577832
C	2.583956	-1.567912	1.818256
C	2.583956	-2.327489	0.589235
C	0.692687	-2.941999	1.818256
C	1.415087	-3.176721	0.589235
C	-1.415087	-1.947701	2.577832
C	-0.692687	-2.941999	1.818256
C	-1.415087	-3.176721	0.589235
C	-2.583956	-1.567912	1.818256

C	-2.583956	-2.327489	0.589235
C	0.000000	2.407490	2.577832
C	-1.168868	2.787279	1.818256
C	-0.722400	3.401789	0.589235
C	1.168868	2.787279	1.818256
C	0.722400	3.401789	0.589235
C	-2.289659	0.743955	2.577832
C	-3.012060	-0.250343	1.818256
C	-3.458528	0.364167	0.589235
C	-2.289659	1.972976	1.818256
C	-3.012060	1.738254	0.589235
C	-1.168868	-0.379788	-3.306166
C	0.000000	-1.229021	-3.306166
C	1.168868	-0.379788	-3.306166
C	-0.722400	0.994299	-3.306166
C	0.722400	0.994299	-3.306166
C	-2.289659	-0.743955	-2.577832
C	-3.012060	0.250343	-1.818256
C	-3.458528	-0.364167	-0.589235
C	-2.289659	-1.972976	-1.818256
C	-3.012060	-1.738254	-0.589235
C	0.000000	-2.407490	-2.577832

C	-1.168868	-2.787279	-1.818256
C	-0.722400	-3.401789	-0.589235
C	1.168868	-2.787279	-1.818256
C	0.722400	-3.401789	-0.589235
C	2.289659	-0.743955	-2.577832
C	2.289659	-1.972976	-1.818256
C	3.012060	-1.738254	-0.589235
C	3.012060	0.250343	-1.818256
C	3.458528	-0.364167	-0.589235
C	-1.415087	1.947701	-2.577832
C	-0.692687	2.941999	-1.818256
C	-1.415087	3.176721	-0.589235
C	-2.583956	1.567912	-1.818256
C	-2.583956	2.327489	-0.589235
C	1.415087	1.947701	-2.577832
C	2.583956	1.567912	-1.818256
C	2.583956	2.327489	-0.589235
C	0.692687	2.941999	-1.818256
C	1.415087	3.176721	-0.589235

Cartesian coordinates of the monomer 1,2,5-oxadiazole optimized at the PBE0-D3/def2-TZVPP theory level:

N	0.000000	1.116228	0.356548
N	0.000000	-1.116228	0.356548
C	0.000000	0.706929	-0.875275
C	0.000000	-0.706929	-0.875275
H	0.000000	-1.405635	-1.695394
H	0.000000	1.405635	-1.695394
O	0.000000	0.000000	1.112803

Cartesian coordinates of the monomer 2,1,3-benzooxadiazole optimized at the PBE0-D3/def2-TZVPP theory level:

N	0.000000	1.124945	1.548009
N	0.000000	-1.124945	1.548009
C	0.000000	-1.444156	-0.913533
H	0.000000	-2.525530	-0.910527
C	0.000000	-0.716366	-2.060938
H	0.000000	-1.225948	-3.016601
C	0.000000	0.716366	-2.060938
H	0.000000	1.225948	-3.016601
C	0.000000	1.444156	-0.913533
H	0.000000	2.525530	-0.910527
C	0.000000	0.712833	0.300450
C	0.000000	-0.712833	0.300450

O	0.000000	0.000000	2.283798

Cartesian coordinates of the monomer 1,2,5-thiadiazole optimized at the PBE0-D3/def2-TZVPP theory level:

N	0.000000	1.234149	0.042158
N	0.000000	-1.234149	0.042158
C	0.000000	0.707090	-1.164806
C	0.000000	-0.707090	-1.164806
H	0.000000	-1.345773	-2.037793
H	0.000000	1.345773	-2.037793
S	0.000000	0.000000	1.091441

Cartesian coordinates of the monomer 2,1,3-benzothiadiazole optimized at the PBE0-D3/def2-TZVPP theory level:

N	0.000000	1.241244	1.220140
N	0.000000	-1.241244	1.220140
C	0.000000	-1.430176	-1.232649
H	0.000000	-2.511998	-1.225535
C	0.000000	-0.710635	-2.391551
H	0.000000	-1.228741	-3.342820
C	0.000000	0.710635	-2.391551
H	0.000000	1.228741	-3.342820

C	0.000000	1.430176	-1.232649
H	0.000000	2.511998	-1.225535
C	0.000000	0.717746	-0.012789
C	0.000000	-0.717746	-0.012789
S	0.000000	0.000000	2.231163

Cartesian coordinates of the monomer 1,2,5-selenadiazole optimized at the PBE0-D3/def2-TZVPP theory level:

N	0.000000	1.301888	-0.381776
N	0.000000	-1.301888	-0.381776
C	0.000000	0.715749	-1.544825
C	0.000000	-0.715749	-1.544825
H	0.000000	-1.320591	-2.445594
H	0.000000	1.320591	-2.445594
Se	0.000000	0.000000	0.846293

Cartesian coordinates of the monomer 2,1,3-benzoselenadiazole optimized at the PBE0-D3/def2-TZVPP theory level:

N	0.000000	1.308494	0.678128
Se	0.000000	0.000000	1.870875
N	0.000000	-1.308494	0.678128
C	0.000000	-1.427895	-1.747284

H	0.000000	-2.509813	-1.739027
C	0.000000	-0.713347	-2.902824
H	0.000000	-1.232305	-3.853720
C	0.000000	0.713347	-2.902824
H	0.000000	1.232305	-3.853720
C	0.000000	1.427895	-1.747284
H	0.000000	2.509813	-1.739027
C	0.000000	0.726182	-0.509730
C	0.000000	-0.726182	-0.509730

Cartesian coordinates of the monomer 1,2,5-telluradiazole optimized at the PBE0-D3/def2-TZVPP theory level:

N	0.000000	1.367047	-0.717340
N	0.000000	-1.367047	-0.717340
C	0.000000	0.723951	-1.840195
C	0.000000	-0.723951	-1.840195
H	0.000000	-1.290013	-2.770444
H	0.000000	1.290013	-2.770444
Te	0.000000	0.000000	0.724346

Cartesian coordinates of the monomer 2,1,3-benzotelluradiazole optimized at the PBE0-D3/def2-TZVPP theory level:

N	0.000000	1.371749	0.247221
N	0.000000	-1.371749	0.247221
C	0.000000	-1.423591	-2.156357
H	0.000000	-2.505679	-2.145967
C	0.000000	-0.714559	-3.311462
H	0.000000	-1.235359	-4.261469
C	0.000000	0.714559	-3.311462
H	0.000000	1.235359	-4.261469
C	0.000000	1.423591	-2.156357
H	0.000000	2.505679	-2.145967
C	0.000000	0.734175	-0.903729
C	0.000000	-0.734175	-0.903729
Te	0.000000	0.000000	1.650238

Cartesian coordinates of the monomer 1,2,4,5-thiatriazole optimized at the PBE0-D3/def2-TZVPP theory level:

N	-1.211925	-0.025503	0.000000
N	1.222355	0.020964	0.000000
C	-0.643700	1.156668	0.000000
H	-1.219108	2.071307	0.000000
N	0.715471	1.201198	0.000000
S	0.000000	-1.086745	0.000000

Cartesian coordinates of the monomer 1,2,4,5-selenatriazole optimized at the PBE0-D3/def2-TZVPP theory level:

N	1.264180	-0.390033	0.000000
N	-1.291387	-0.479484	0.000000
C	0.663421	-1.538492	0.000000
H	1.215818	-2.469436	0.000000
Se	0.000000	0.851491	0.000000
N	-0.715128	-1.594813	0.000000

Cartesian coordinates of the monomer 1,2,4,5-telluratriazole optimized at the PBE0-D3/def2-TZVPP theory level:

N	1.311614	-0.720822	0.000000
N	-1.357663	-0.857981	0.000000
C	0.682888	-1.843611	0.000000
H	1.210753	-2.791352	0.000000
N	-0.712248	-1.913613	0.000000
Te	0.000000	0.736537	0.000000

Cartesian coordinates of the complex S-1 optimized at the PBE0-D3/6-31G(d) theory level:

C	0.006416	-0.079238	-3.448825

C	-0.838372	1.003413	-3.001134
C	-1.950225	0.429649	-2.276643
C	-0.587967	-1.326424	-3.005048
C	-1.809963	-1.026237	-2.305059
C	1.388787	0.076701	-3.460030
C	2.244873	-1.005761	-3.024543
C	3.353929	-0.427879	-2.296695
C	1.978498	1.324914	-3.014669
C	3.194458	1.013361	-2.297070
C	-0.272073	2.206009	-2.582986
C	1.165756	2.371618	-2.590702
C	1.532566	3.143112	-1.420171
C	-0.794066	2.874504	-1.419488
C	0.320344	3.456599	-0.694472
C	-2.478887	1.092194	-1.187621
C	-1.857081	2.301031	-0.724693
C	-1.857101	2.301155	0.724255
C	-3.069980	0.338258	-0.000075
C	-2.478924	1.092396	1.187376
C	0.241023	-2.374997	-2.597237
C	-0.116218	-3.133296	-1.423094
C	1.095949	-3.450005	-0.695461

C	1.675845	-2.200891	-2.591771
C	2.207823	-2.871106	-1.420324
C	-2.133001	-1.734354	-1.152376
C	-2.780666	-1.145919	0.000061
C	-2.133042	-1.734173	1.152599
C	-1.275610	-2.818828	-0.719465
C	-1.275631	-2.818712	0.719884
C	1.388704	0.077279	3.460030
C	1.978426	1.325416	3.014474
C	3.194402	1.013743	2.296959
C	2.244802	-1.005253	3.024751
C	3.353874	-0.427497	2.296828
C	0.006332	-0.078660	3.448820
C	-0.588044	-1.325921	3.005245
C	-1.810035	-1.025859	2.305186
C	-0.838442	1.003918	3.000926
C	-1.950284	0.430033	2.276514
C	1.165695	2.372048	2.590314
C	-0.272132	2.206442	2.582591
C	-0.794098	2.874743	1.418968
C	1.532529	3.143347	1.419663
C	0.320326	3.456713	0.693878

C	3.545963	1.753363	1.171202
C	2.699384	2.839439	0.724085
C	2.699402	2.839319	-0.724511
C	4.073896	1.085461	-0.000050
C	3.545992	1.753168	-1.171427
C	1.675781	-2.200453	2.592165
C	2.207789	-2.870865	1.420843
C	1.095931	-3.449886	0.696052
C	0.240955	-2.374559	2.597626
C	-0.116259	-3.133057	1.423602
C	3.862769	-1.069086	1.172162
C	4.222273	-0.298199	0.000066
C	3.862796	-1.069281	-1.171909
C	3.275684	-2.314973	0.725030
C	3.275703	-2.315095	-0.724580
N	-4.574612	0.405628	-0.000091
N	-6.819777	-0.424489	-0.000053
C	-5.432254	1.397511	0.000182
C	-6.753236	0.886591	0.000094
H	-7.654633	1.491203	-0.000558
H	-5.095907	2.426967	0.000004
S	-5.322577	-1.128003	-0.000016

Cartesian coordinates of the complex S-2 optimized at the PBE0-D3/6-31G(d) theory level:

C	0.460949	-0.171758	-3.448740
C	-0.556029	0.750476	-3.000293
C	-1.555456	-0.004547	-2.276849
C	0.087582	-1.502148	-3.005463
C	-1.168391	-1.414906	-2.307165
C	1.796742	0.217616	-3.460248
C	2.825098	-0.702976	-3.025341
C	3.819283	0.055475	-2.297122
C	2.165238	1.547995	-3.014911
C	3.416598	1.448448	-2.297357
C	-0.202672	2.032185	-2.583492
C	1.185854	2.440777	-2.591186
C	1.415895	3.263234	-1.420349
C	-0.830145	2.600239	-1.418971
C	0.168200	3.364252	-0.694192
C	-2.191397	0.556689	-1.187634
C	-1.778262	1.851637	-0.724571
C	-1.778147	1.851682	0.724905
C	-2.654513	-0.286686	0.000303

C	-2.191220	0.556766	1.188113
C	1.083908	-2.394070	-2.597895
C	0.861719	-3.202083	-1.423454
C	2.110107	-3.307467	-0.695779
C	2.467770	-1.977445	-2.592131
C	3.106543	-2.547078	-1.420596
C	-1.363016	-2.165210	-1.151565
C	-2.095260	-1.692688	0.000292
C	-1.362863	-2.165162	1.152074
C	-0.333654	-3.089299	-0.719204
C	-0.333550	-3.089260	0.719604
C	1.797273	0.217817	3.460124
C	2.165702	1.548170	3.014654
C	3.416951	1.448580	2.296913
C	2.825561	-0.702800	3.025112
C	3.819635	0.055608	2.296697
C	0.461479	-0.171559	3.448838
C	0.088041	-1.501973	3.005696
C	-1.168044	-1.414776	2.307591
C	-0.555567	0.750651	3.000498
C	-1.555108	-0.004411	2.277255
C	1.186254	2.440929	2.591029

C	-0.202274	2.032338	2.583573
C	-0.829925	2.600327	1.419114
C	1.416115	3.263318	1.420109
C	0.168308	3.364295	0.694136
C	3.637172	2.237429	1.171092
C	2.617509	3.163016	0.724156
C	2.617396	3.162973	-0.724575
C	4.271228	1.669622	-0.000293
C	3.636991	2.237360	-1.171613
C	2.468165	-1.977292	2.592030
C	3.106759	-2.546993	1.420431
C	2.110212	-3.307425	0.695812
C	1.084304	-2.393919	2.598031
C	0.861934	-3.202002	1.423674
C	4.430364	-0.489601	1.171781
C	4.652860	0.331292	-0.000284
C	4.430184	-0.489669	-1.172268
C	4.064417	-1.817459	0.724646
C	4.064307	-1.817502	-0.725001
N	-4.141120	-0.496608	0.000381
N	-6.184401	-1.780629	-0.000140
C	-7.666441	0.174918	-0.000217

H	-8.566582	-0.429699	-0.000386
C	-7.692147	1.540788	-0.000169
H	-8.642981	2.064368	-0.000286
C	-6.488645	2.309002	0.000023
H	-6.566043	3.392236	0.000074
C	-5.244411	1.734851	0.000175
H	-4.336628	2.325483	0.000329
C	-5.195101	0.322747	0.000151
C	-6.398741	-0.467703	-0.000055
S	-4.592931	-2.135347	0.000043

Cartesian coordinates of the complex Se-1 optimized at the PBE0-D3/6-31G(d) theory level:

C	0.220660	-0.017466	-3.445242
C	-0.531813	1.133188	-2.999044
C	-1.687716	0.657784	-2.274328
C	-0.473507	-1.210843	-3.001622
C	-1.661836	-0.808638	-2.294104
C	1.610774	0.022445	-3.458305
C	2.373409	-1.128118	-3.021360
C	3.528041	-0.645346	-2.296268
C	2.303740	1.216838	-3.015092

C	3.488591	0.804405	-2.296885
C	0.134668	2.284778	-2.583900
C	1.581295	2.327871	-2.591115
C	2.012093	3.066497	-1.421153
C	-0.328277	2.994843	-1.420451
C	0.830579	3.481001	-0.695434
C	-2.153842	1.359470	-1.183812
C	-1.436025	2.514016	-0.725071
C	-1.435961	2.514472	0.723695
C	-2.820793	0.667354	-0.000050
C	-2.153742	1.360215	1.183231
C	0.264282	-2.324762	-2.593607
C	-0.154597	-3.053187	-1.422741
C	1.027122	-3.469370	-0.694521
C	1.709383	-2.273555	-2.590953
C	2.183268	-2.985602	-1.419707
C	-2.049538	-1.495473	-1.155328
C	-2.676794	-0.859015	0.000447
C	-2.049425	-1.494734	1.156561
C	-1.283417	-2.638481	-0.719906
C	-1.283352	-2.638024	0.721794
C	1.611094	0.024648	3.458235

C	2.304019	1.218760	3.014197
C	3.488804	0.805868	2.296145
C	2.373692	-1.126191	3.021954
C	3.528256	-0.643884	2.296446
C	0.220980	-0.015271	3.445326
C	-0.473228	-1.208926	3.002530
C	-1.661627	-0.807180	2.294876
C	-0.531535	1.135093	2.998461
C	-1.687504	0.659226	2.274155
C	1.581533	2.329521	2.589577
C	0.134905	2.286420	2.582521
C	-0.328148	2.995745	1.418663
C	2.012221	3.067402	1.419105
C	0.830640	3.481441	0.693230
C	3.901718	1.513361	1.170706
C	3.149253	2.666341	0.723438
C	3.149188	2.665880	-0.725334
C	4.370710	0.802393	-0.000412
C	3.901608	1.512614	-1.171935
C	1.709621	-2.271899	2.592334
C	2.183399	-2.984695	1.421501
C	1.027187	-3.468926	0.696732

C	0.264522	-2.323106	2.595161
C	-0.154466	-3.052272	1.424792
C	3.981887	-1.326450	1.172067
C	4.405001	-0.588498	0.000029
C	3.981777	-1.327197	-1.171497
C	3.294127	-2.519602	0.725464
C	3.294060	-2.520064	-0.724070
N	-4.291872	0.809465	-0.000034
N	-6.643068	0.006110	0.000243
C	-5.140585	1.789352	0.000050
C	-6.489321	1.294274	0.000054
H	-7.345409	1.967745	0.000277
H	-4.823156	2.826275	0.000134
Se	-5.035683	-0.913632	-0.000096

Cartesian coordinates of the complex Se-2 optimized at the PBE0-D3/6-31G(d) theory level:

C	0.629502	-0.100576	-3.445675
C	-0.313374	0.899556	-2.998757
C	-1.368998	0.228151	-2.275035
C	0.155418	-1.397573	-3.002477

C	-1.085917	-1.210615	-2.296973
C	1.991196	0.182921	-3.458603
C	2.944302	-0.815819	-3.022159
C	3.995836	-0.137696	-2.296429
C	2.463526	1.480334	-3.014950
C	3.702455	1.282509	-2.296747
C	0.140463	2.150335	-2.584032
C	1.557000	2.446982	-2.591000
C	1.851316	3.249343	-1.420706
C	-0.439854	2.766586	-1.419838
C	0.615287	3.448956	-0.694781
C	-1.952703	0.835455	-1.184012
C	-1.445014	2.096534	-0.725024
C	-1.445031	2.096742	0.724404
C	-2.494193	0.036756	-0.000025
C	-1.952750	0.835784	1.183706
C	1.078055	-2.364686	-2.594670
C	0.793980	-3.155174	-1.423554
C	2.030364	-3.357506	-0.695203
C	2.491411	-2.059840	-2.591309
C	3.083383	-2.677727	-1.420138
C	-1.344326	-1.952789	-1.155023

C	-2.065116	-1.433505	0.000129
C	-1.344376	-1.952471	1.155476
C	-0.389666	-2.945374	-0.720173
C	-0.389681	-2.945159	0.720938
C	1.991101	0.183854	3.458591
C	2.463443	1.481151	3.014594
C	3.702391	1.283129	2.296484
C	2.944215	-0.815000	3.022436
C	3.995770	-0.137076	2.296555
C	0.629409	-0.099646	3.445691
C	0.155330	-1.396762	3.002837
C	-1.085963	-1.209981	2.297218
C	-0.313457	0.900369	2.998479
C	-1.369052	0.228779	2.274909
C	1.556935	2.447691	2.590365
C	0.140392	2.151042	2.583442
C	-0.439889	2.766971	1.419061
C	1.851279	3.249731	1.419860
C	0.615269	3.449154	0.693854
C	3.984600	2.052350	1.171120
C	3.041041	3.055058	0.724039
C	3.041058	3.054860	-0.724799

C	4.571528	1.435415	-0.000141
C	3.984635	2.052034	-1.171585
C	2.491337	-2.059141	2.591913
C	3.083341	-2.677344	1.420924
C	2.030339	-3.357311	0.696138
C	1.077983	-2.363978	2.595306
C	0.793937	-3.154807	1.424407
C	4.562478	-0.728911	1.171994
C	4.849435	0.072059	0.000046
C	4.562515	-0.729228	-1.171693
C	4.095235	-2.024357	0.725094
C	4.095260	-2.024553	-0.724457
N	-3.963413	-0.095247	-0.000023
Se	-4.395085	-1.904256	-0.000008
N	-6.125023	-1.347101	0.000033
C	-7.475242	0.676029	0.000048
H	-8.396468	0.103402	0.000054
C	-7.458224	2.039054	0.000033
H	-8.390556	2.595222	0.000051
C	-6.226438	2.764763	-0.000026
H	-6.263650	3.850381	-0.000030
C	-5.007474	2.143818	-0.000069

H	-4.079435	2.703359	-0.000145
C	-4.994582	0.727707	-0.000021
C	-6.235420	-0.035894	0.000038

Cartesian coordinates of the complex Te-1 optimized at the PBE0-D3/SDD theory level:

C	0.443875	0.016243	-3.465335
C	-0.261716	1.205957	-3.015305
C	-1.445019	0.781822	-2.286380
C	-0.305796	-1.151556	-3.017903
C	-1.478461	-0.693555	-2.300404
C	1.841085	-0.006234	-3.478451
C	2.555570	-1.197178	-3.037301
C	3.739057	-0.764718	-2.309494
C	2.591781	1.162671	-3.033079
C	3.763552	0.694296	-2.309434
C	0.459374	2.332219	-2.598842
C	1.915517	2.309673	-2.605760
C	2.381462	3.032426	-1.428410
C	0.025465	3.064260	-1.426964
C	1.211859	3.501108	-0.698114
C	-1.878814	1.502500	-1.188197

C	-1.109371	2.632454	-0.728512
C	-1.109322	2.632539	0.728272
C	-2.599872	0.849460	0.000029
C	-1.878730	1.502634	1.188132
C	0.383524	-2.302252	-2.606031
C	-0.071314	-3.016754	-1.430451
C	1.098285	-3.489064	-0.698669
C	1.839029	-2.318903	-2.606674
C	2.282775	-3.055917	-1.428292
C	-1.906238	-1.369791	-1.165269
C	-2.547800	-0.709143	0.000117
C	-1.906165	-1.369658	1.165538
C	-1.187216	-2.547784	-0.725551
C	-1.187170	-2.547701	0.725908
C	1.841312	-0.005835	3.478321
C	2.591980	1.163019	3.032766
C	3.763703	0.694560	2.309099
C	2.555768	-1.196830	3.037261
C	3.739208	-0.764453	2.309328
C	0.444101	0.016641	3.465294
C	-0.305599	-1.151209	3.018044
C	-1.478311	-0.693290	2.300567

C	-0.261519	1.206304	3.015175
C	-1.444868	0.782085	2.286372
C	1.915688	2.309971	2.605360
C	0.459544	2.332517	2.598536
C	0.025558	3.064424	1.426601
C	2.381556	3.032590	1.427897
C	1.211905	3.501188	0.697623
C	4.210399	1.386764	1.178474
C	3.505290	2.579427	0.728710
C	3.505242	2.579344	-0.729245
C	4.648822	0.650783	-0.000194
C	4.210322	1.386629	-1.178918
C	1.839198	-2.318604	2.606808
C	2.282868	-3.055753	1.428482
C	1.098330	-3.488984	0.698986
C	0.383693	-2.301953	2.606258
C	-0.071221	-3.016589	1.430789
C	4.163700	-1.469917	1.178844
C	4.623288	-0.747192	-0.000113
C	4.163622	-1.470052	-1.178957
C	3.419601	-2.638708	0.729248
C	3.419554	-2.638791	-0.729179

N	-4.058945	1.088344	0.000049
N	-6.570697	0.510729	0.000399
C	-4.827224	2.139209	-0.000046
C	-6.246546	1.781132	0.000392
H	-7.009236	2.556762	0.000407
H	-4.453473	3.157592	-0.000133
Te	-4.974978	-0.824592	0.000193

Cartesian coordinates of the complex Te-2 optimized at the PBE0-D3/SDD theory level:

C	0.806011	-0.057085	-3.465174
C	-0.094611	0.992460	-3.014691
C	-1.188389	0.370938	-2.286536
C	0.269143	-1.337012	-3.018379
C	-0.965526	-1.088085	-2.301906
C	2.186314	0.162074	-3.478433
C	3.095899	-0.887490	-3.037248
C	4.186976	-0.257070	-2.309489
C	2.724008	1.443009	-3.033326
C	3.959103	1.184168	-2.309557
C	0.421534	2.226287	-2.599210
C	1.859675	2.455795	-2.606037

C	2.193980	3.247980	-1.428565
C	-0.131920	2.870833	-1.426546
C	0.961008	3.507161	-0.698236
C	-1.742011	1.004752	-1.188237
C	-1.174035	2.248280	-0.728816
C	-1.174017	2.248463	0.728287
C	-2.347165	0.237280	0.000005
C	-1.741982	1.005050	1.188037
C	1.147424	-2.351251	-2.606039
C	0.823233	-3.133515	-1.430420
C	2.056799	-3.396916	-0.698495
C	2.583799	-2.115940	-2.606373
C	3.148537	-2.765356	-1.428127
C	-1.267175	-1.826421	-1.164492
C	-2.007838	-1.284399	0.000191
C	-1.267143	-1.826128	1.164990
C	-0.356444	-2.863935	-0.725194
C	-0.356425	-2.863753	0.725927
C	2.186406	0.162947	3.478338
C	2.724088	1.443770	3.032894
C	3.959164	1.184747	2.309158
C	3.095981	-0.886728	3.037392

C	4.187038	-0.256490	2.309446
C	0.806103	-0.056215	3.465170
C	0.269223	-1.336254	3.018713
C	-0.965465	-1.087508	2.302210
C	-0.094531	0.993216	3.014447
C	-1.188330	0.371512	2.286479
C	1.859744	2.456449	2.605374
C	0.421603	2.226939	2.598642
C	-0.131882	2.871189	1.425831
C	2.194018	3.248338	1.427693
C	0.961026	3.507335	0.697332
C	4.279715	1.943509	1.178426
C	3.379307	2.996541	0.728575
C	3.379289	2.996359	-0.729414
C	4.838868	1.294387	-0.000225
C	4.279684	1.943214	-1.179024
C	2.583869	-2.115287	2.606841
C	3.148576	-2.764999	1.428742
C	2.056818	-3.396741	0.699298
C	1.147493	-2.350596	2.606603
C	0.823272	-3.133156	1.431189
C	4.727224	-0.878106	1.179021

C	5.055417	-0.087046	-0.000055
C	4.727193	-0.878402	-1.178923
C	4.196113	-2.157839	0.729409
C	4.196094	-2.158021	-0.728975
N	-3.822421	0.199341	0.000037
Te	-4.376192	-1.824651	0.000297
N	-6.185684	-0.856700	-0.000011
C	-7.284474	1.321230	-0.000170
H	-8.256181	0.840226	-0.000199
C	-7.135320	2.685136	-0.000271
H	-8.007235	3.331162	-0.000380
C	-5.826681	3.284952	-0.000262
H	-5.753118	4.368779	-0.000360
C	-4.672093	2.535193	-0.000162
H	-3.698415	3.008815	-0.000180
C	-4.783615	1.111861	-0.000035
C	-6.120761	0.470123	-0.000038

Cartesian coordinates of the complex S-1 optimized at the PBE0/6-31G(d) theory level:

C	0.007230	-0.077782	-3.446542
C	-0.834928	1.005429	-2.999051

C	-1.947027	0.434064	-2.275352
C	-0.588844	-1.322893	-3.003076
C	-1.809386	-1.021079	-2.303816
C	1.390043	0.075641	-3.458136
C	2.243606	-1.007564	-3.023262
C	3.352769	-0.432061	-2.295955
C	1.981563	1.321838	-3.013190
C	3.196096	1.008385	-2.296299
C	-0.266328	2.207040	-2.581164
C	1.170586	2.370087	-2.589099
C	1.538493	3.140226	-1.419506
C	-0.786758	2.875795	-1.418693
C	0.327803	3.455470	-0.694354
C	-2.476738	1.097875	-1.186609
C	-1.851362	2.304733	-0.724148
C	-1.851373	2.304795	0.723922
C	-3.071808	0.345051	-0.000039
C	-2.476757	1.097976	1.186478
C	0.238383	-2.373163	-2.595810
C	-0.119836	-3.129971	-1.422617
C	1.090710	-3.448874	-0.695668
C	1.672393	-2.201814	-2.590420

C	2.202804	-2.872533	-1.419978
C	-2.134296	-1.728829	-1.151043
C	-2.784310	-1.140543	0.000023
C	-2.134304	-1.728730	1.151144
C	-1.278813	-2.813347	-0.718832
C	-1.278822	-2.813293	0.719044
C	1.390001	0.075926	3.458139
C	1.981523	1.322086	3.013099
C	3.196067	1.008574	2.296250
C	2.243571	-1.007314	3.023368
C	3.352743	-0.431872	2.296027
C	0.007187	-0.077499	3.446540
C	-0.588882	-1.322645	3.003171
C	-1.809415	-1.020889	2.303870
C	-0.834966	1.005672	2.998947
C	-1.947055	0.434250	2.275279
C	1.170550	2.370299	2.588911
C	-0.266364	2.207250	2.580970
C	-0.786777	2.875911	1.418434
C	1.538472	3.140343	1.419260
C	0.327793	3.455526	0.694064
C	3.548962	1.747684	1.170349

C	2.705001	2.834466	0.723627
C	2.705011	2.834407	-0.723832
C	4.075330	1.079407	-0.000022
C	3.548977	1.747587	-1.170454
C	1.672363	-2.201599	2.590617
C	2.202788	-2.872414	1.420236
C	1.090702	-3.448815	0.695961
C	0.238353	-2.372947	2.596004
C	-0.119854	-3.129855	1.422871
C	3.860648	-1.074432	1.171232
C	4.221157	-0.304696	0.000036
C	3.860660	-1.074528	-1.171101
C	3.271805	-2.318279	0.724371
C	3.271814	-2.318340	-0.724146
N	-4.581717	0.410396	-0.000049
N	-6.825436	-0.430440	0.000151
C	-5.446026	1.397218	0.000052
C	-6.764392	0.880606	0.000096
H	-7.668284	1.481805	-0.000393
H	-5.118075	2.429515	-0.000130
S	-5.324674	-1.125569	-0.000078

Cartesian coordinates of the complex S-2 optimized at the PBE0/6-31G(d) theory level:

C	0.463563	-0.168831	-3.446498
C	-0.548530	0.757095	-2.998309
C	-1.550710	0.007276	-2.275665
C	0.084632	-1.496365	-3.003435
C	-1.169899	-1.403697	-2.305800
C	1.801249	0.214574	-3.458372
C	2.824753	-0.709869	-3.023859
C	3.821436	0.043686	-2.296233
C	2.175400	1.542318	-3.013563
C	3.425353	1.437419	-2.296627
C	-0.189308	2.037264	-2.581861
C	1.199822	2.439492	-2.589809
C	1.433278	3.260282	-1.420025
C	-0.813823	2.607506	-1.418469
C	0.187027	3.366571	-0.694495
C	-2.186707	0.571296	-1.186804
C	-1.766427	1.863951	-0.724364
C	-1.766372	1.864120	0.724218
C	-2.656503	-0.269957	0.000204
C	-2.186633	0.571578	1.186988

C	1.077074	-2.392833	-2.596165
C	0.851443	-3.198720	-1.422582
C	2.098251	-3.309854	-0.695479
C	2.461735	-1.982838	-2.590465
C	3.097416	-2.554771	-1.419796
C	-1.368489	-2.153153	-1.150106
C	-2.101984	-1.678920	0.000335
C	-1.368446	-2.152917	1.150844
C	-0.343571	-3.080191	-0.718232
C	-0.343531	-3.080028	0.719099
C	1.801479	0.215359	3.458345
C	2.175605	1.543004	3.013213
C	3.425507	1.437939	2.296213
C	2.824952	-0.709184	3.023971
C	3.821587	0.044206	2.296108
C	0.463792	-0.168048	3.446645
C	0.084826	-1.495683	3.003906
C	-1.169759	-1.403178	2.306344
C	-0.548328	0.757782	2.998319
C	-1.550566	0.007801	2.275925
C	1.200002	2.440086	2.589322
C	-0.189130	2.037861	2.581563

C	-0.813719	2.607836	1.418085
C	1.433378	3.260610	1.419335
C	0.187078	3.366735	0.693866
C	3.649289	2.225779	1.170150
C	2.634554	3.155105	0.723428
C	2.634503	3.154939	-0.724177
C	4.280398	1.655482	-0.000260
C	3.649208	2.225513	-1.170757
C	2.461902	-1.982248	2.590890
C	3.097508	-2.554448	1.420310
C	2.098295	-3.309694	0.696233
C	1.077241	-2.392245	2.596776
C	0.851533	-3.198395	1.423393
C	4.430082	-0.503903	1.171145
C	4.656029	0.315290	-0.000121
C	4.430005	-0.504169	-1.171185
C	4.058568	-1.829121	0.724426
C	4.058522	-1.829286	-0.724141
N	-4.150065	-0.482135	0.000254
N	-6.185221	-1.784489	-0.000045
C	-7.684987	0.155490	-0.000472
H	-8.578529	-0.459244	-0.000504

C	-7.726080	1.520855	-0.000662
H	-8.682523	2.034493	-0.000862
C	-6.530311	2.300889	-0.000587
H	-6.618542	3.383504	-0.000696
C	-5.280179	1.739328	-0.000300
H	-4.379586	2.341353	-0.000189
C	-5.213600	0.327025	-0.000114
C	-6.410170	-0.473836	-0.000313
S	-4.591056	-2.124469	0.000238

Cartesian coordinates of the complex Se-1 optimized at the PBE0/6-31G(d) theory level:

C	0.223081	-0.017246	-3.442877
C	-0.529701	1.131636	-2.996607
C	-1.684480	0.655453	-2.272910
C	-0.469533	-1.210340	-2.999841
C	-1.657485	-0.809976	-2.293297
C	1.613350	0.023817	-3.456445
C	2.376474	-1.125361	-3.020448
C	3.529728	-0.642078	-2.295735
C	2.304795	1.217814	-3.013394
C	3.489171	0.806563	-2.295994

C	0.135909	2.283805	-2.581594
C	1.581310	2.328279	-2.589058
C	2.011144	3.066366	-1.419877
C	-0.327338	2.992527	-1.419045
C	0.830189	3.479210	-0.694616
C	-2.153574	1.357287	-1.182703
C	-1.435241	2.511163	-0.724040
C	-1.435189	2.511246	0.723824
C	-2.823760	0.664721	0.000048
C	-2.153489	1.357424	1.182670
C	0.269377	-2.323985	-2.592782
C	-0.148477	-3.052194	-1.423000
C	1.032549	-3.467436	-0.695554
C	1.713259	-2.271560	-2.590143
C	2.187429	-2.982991	-1.420057
C	-2.045935	-1.497528	-1.154272
C	-2.676906	-0.862770	0.000124
C	-2.045861	-1.497401	1.154551
C	-1.277896	-2.638946	-0.719982
C	-1.277846	-2.638865	0.720334
C	1.613595	0.024206	3.456291
C	2.305009	1.218153	3.013057

C	3.489333	0.806821	2.295618
C	2.376687	-1.125022	3.020369
C	3.529890	-0.641820	2.295520
C	0.223325	-0.016859	3.442825
C	-0.469321	-1.210002	2.999973
C	-1.657325	-0.809717	2.293468
C	-0.529488	1.131974	2.996481
C	-1.684319	0.655710	2.272920
C	1.581495	2.328571	2.588648
C	0.136093	2.284096	2.581291
C	-0.327237	2.992689	1.418696
C	2.011245	3.066526	1.419353
C	0.830239	3.479289	0.694129
C	3.901700	1.515056	1.170178
C	3.148876	2.666659	0.723546
C	3.148825	2.666577	-0.724107
C	4.371012	0.805351	-0.000219
C	3.901618	1.514924	-1.170662
C	1.713442	-2.271269	2.590240
C	2.187529	-2.982831	1.420201
C	1.032598	-3.467357	0.695834
C	0.269560	-2.323694	2.592987

C	-0.148377	-3.052034	1.423317
C	3.984261	-1.323825	1.170833
C	4.406346	-0.585720	-0.000142
C	3.984178	-1.323957	-1.171003
C	3.298051	-2.516527	0.724233
C	3.298000	-2.516608	-0.724220
N	-4.299129	0.807772	0.000096
N	-6.654678	0.007209	0.000367
C	-5.149243	1.787680	-0.000001
C	-6.498344	1.295263	0.000115
H	-7.353576	1.969954	0.000076
H	-4.833892	2.825407	-0.000172
Se	-5.051228	-0.912524	0.000428

Cartesian coordinates of the complex Se-2 optimized at the PBE0/6-31G(d) theory level:

C	0.633144	-0.099363	-3.443101
C	-0.307259	0.901285	-2.996197
C	-1.363474	0.232256	-2.273532
C	0.157417	-1.394465	-3.000348

C	-1.082775	-1.205920	-2.295880
C	1.995475	0.181933	-3.456634
C	2.946311	-0.817559	-3.020910
C	3.998050	-0.141612	-2.295741
C	2.469583	1.477575	-3.013496
C	3.707293	1.277976	-2.296047
C	0.148744	2.151342	-2.581990
C	1.564499	2.445712	-2.589389
C	1.859883	3.246863	-1.420118
C	-0.430099	2.767735	-1.418907
C	0.625221	3.447965	-0.694738
C	-1.949341	0.840860	-1.183104
C	-1.437286	2.100033	-0.724446
C	-1.437273	2.100209	0.723958
C	-2.497127	0.043110	0.000017
C	-1.949320	0.841147	1.182929
C	1.078662	-2.363213	-2.593173
C	0.793648	-3.152233	-1.422972
C	2.028600	-3.356717	-0.695351
C	2.491320	-2.060948	-2.589896
C	3.081894	-2.679335	-1.419748
C	-1.343304	-1.947705	-1.153450

C	-2.066347	-1.428392	0.000186
C	-1.343291	-1.947430	1.153940
C	-0.389719	-2.940644	-0.719465
C	-0.389707	-2.940469	0.720178
C	1.995533	0.182768	3.456552
C	2.469634	1.478303	3.013091
C	3.707332	1.278531	2.295670
C	2.946361	-0.816829	3.021051
C	3.998089	-0.141057	2.295701
C	0.633203	-0.098532	3.443108
C	0.157468	-1.393741	3.000677
C	-1.082735	-1.205365	2.296181
C	-0.307209	0.902009	2.995980
C	-1.363436	0.232806	2.273495
C	1.564543	2.446338	2.588767
C	0.148788	2.151965	2.581462
C	-0.430075	2.768078	1.418240
C	1.859907	3.247206	1.419296
C	0.625233	3.448133	0.693889
C	3.990775	2.047403	1.170141
C	3.049581	3.050838	0.723441
C	3.049568	3.050663	-0.724235

C	4.576235	1.430037	-0.000214
C	3.990755	2.047120	-1.170709
C	2.491364	-2.060322	2.590346
C	3.081918	-2.678993	1.420338
C	2.028611	-3.356547	0.696122
C	1.078706	-2.362586	2.593719
C	0.793671	-3.151891	1.423715
C	4.563946	-0.733875	1.170996
C	4.851858	0.066037	-0.000052
C	4.563927	-0.734158	-1.170902
C	4.094983	-2.027527	0.724419
C	4.094971	-2.027702	-0.724004
N	-3.972251	-0.089173	0.000044
Se	-4.405125	-1.900139	0.000388
N	-6.133488	-1.349701	0.000250
C	-7.492135	0.665156	-0.000066
H	-8.409538	0.086013	0.000032
C	-7.484628	2.028123	-0.000297
H	-8.420482	2.578722	-0.000389
C	-6.256785	2.760117	-0.000428
H	-6.299670	3.845740	-0.000615
C	-5.034037	2.146099	-0.000331

H	-4.110794	2.713909	-0.000437
C	-5.009455	0.729163	-0.000086
C	-6.247403	-0.039023	0.000049

Cartesian coordinates of the complex Te-1 optimized at the PBE0/SDD theory level:

C	0.446549	0.016048	-3.463602
C	-0.258832	1.204922	-3.013657
C	-1.441755	0.780984	-2.285519
C	-0.302490	-1.151225	-3.016285
C	-1.474885	-0.693964	-2.299569
C	1.844078	-0.006193	-3.477304
C	2.558547	-1.196459	-3.036626
C	3.741410	-0.763948	-2.309221
C	2.594275	1.162325	-3.032335
C	3.765656	0.694411	-2.309164
C	0.462241	2.331553	-2.597652
C	1.917631	2.309436	-2.604859
C	2.383248	3.031928	-1.428174
C	0.028377	3.063076	-1.426550
C	1.214111	3.500004	-0.698219

C	-1.878052	1.502683	-1.187793
C	-1.107116	2.631748	-0.728265
C	-1.107069	2.631833	0.728019
C	-2.603098	0.849980	0.000024
C	-1.877971	1.502818	1.187720
C	0.387272	-2.302081	-2.604959
C	-0.067149	-3.016145	-1.430066
C	1.101893	-3.488171	-0.698792
C	1.842023	-2.318535	-2.605806
C	2.285756	-3.055149	-1.428080
C	-1.904208	-1.370755	-1.164270
C	-2.549162	-0.710788	0.000113
C	-1.904137	-1.370621	1.164532
C	-1.183595	-2.547743	-0.725108
C	-1.183550	-2.547659	0.725461
C	1.844297	-0.005792	3.477176
C	2.594467	1.162674	3.032026
C	3.765802	0.694677	2.308834
C	2.558738	-1.196110	3.036589
C	3.741555	-0.763683	2.309060
C	0.446767	0.016447	3.463559
C	-0.302300	-1.150877	3.016423

C	-1.474740	-0.693699	2.299726
C	-0.258642	1.205270	3.013523
C	-1.441608	0.781247	2.285504
C	1.917795	2.309735	2.604460
C	0.462405	2.331852	2.597343
C	0.028467	3.063241	1.426183
C	2.383338	3.032092	1.427663
C	1.214155	3.500085	0.697728
C	4.212500	1.387141	1.177932
C	3.507515	2.579121	0.728370
C	3.507469	2.579037	-0.728901
C	4.650873	0.651572	-0.000190
C	4.212426	1.387006	-1.178370
C	1.842186	-2.318235	2.605943
C	2.285846	-3.054984	1.428274
C	1.101936	-3.488090	0.699110
C	0.387435	-2.301781	2.605185
C	-0.067060	-3.015980	1.430403
C	4.166336	-1.469258	1.178317
C	4.625549	-0.746752	-0.000109
C	4.166261	-1.469394	-1.178423
C	3.422825	-2.637653	0.728919

C	3.422779	-2.637737	-0.728844
N	-4.065673	1.088805	0.000041
N	-6.581360	0.511038	0.000429
C	-4.836654	2.138810	-0.000062
C	-6.256153	1.781209	0.000432
H	-7.018566	2.557362	0.000451
H	-4.466299	3.158684	-0.000150
Te	-4.987717	-0.824384	0.000187

Cartesian coordinates of the complex Te-2 optimized at the PBE0/SDD theory level:

C	0.810030	-0.055914	-3.463413
C	-0.088695	0.994133	-3.012885
C	-1.183195	0.374643	-2.285630
C	0.271813	-1.334380	-3.016825
C	-0.962061	-1.084176	-2.301075
C	2.190938	0.161437	-3.477310
C	3.098821	-0.888821	-3.036585
C	4.190239	-0.260113	-2.309243
C	2.730174	1.441071	-3.032607
C	3.964409	1.180773	-2.309311
C	0.429317	2.227471	-2.597906

C	1.866969	2.455149	-2.605106
C	2.202189	3.246478	-1.428312
C	-0.123004	2.872219	-1.426022
C	0.970172	3.506841	-0.698305
C	-1.739026	1.009849	-1.187846
C	-1.167061	2.251756	-0.728537
C	-1.167048	2.251931	0.728013
C	-2.350162	0.243361	-0.000011
C	-1.739005	1.010134	1.187630
C	1.149022	-2.349997	-2.604989
C	0.824025	-3.131213	-1.430040
C	2.056576	-3.396359	-0.698632
C	2.584990	-2.116856	-2.605546
C	3.148674	-2.766727	-1.427945
C	-1.266004	-1.822632	-1.163448
C	-2.009666	-1.280806	0.000170
C	-1.265983	-1.822351	1.163905
C	-0.355581	-2.860231	-0.724739
C	-0.355569	-2.860058	0.725431
C	2.191000	0.162274	3.477231
C	2.730227	1.441800	3.032209
C	3.964450	1.181328	2.308955

C	3.098874	-0.888091	3.036743
C	4.190279	-0.259558	2.309229
C	0.810092	-0.055081	3.463410
C	0.271865	-1.333655	3.017140
C	-0.962020	-1.083622	2.301350
C	-0.088642	0.994858	3.012645
C	-1.183154	0.375193	2.285558
C	1.867015	2.455776	2.604481
C	0.429363	2.228096	2.597359
C	-0.122978	2.872560	1.425330
C	2.202214	3.246821	1.427489
C	0.970185	3.507008	0.697441
C	4.286111	1.939863	1.177937
C	3.387532	2.993571	0.728274
C	3.387520	2.993396	-0.729057
C	4.844134	1.290304	-0.000199
C	4.286090	1.939580	-1.178481
C	2.585036	-2.116229	2.606009
C	3.148700	-2.766384	1.428554
C	2.056589	-3.396191	0.699411
C	1.149067	-2.349371	2.605533
C	0.824050	-3.130869	1.430777

C	4.729828	-0.882026	1.178524
C	5.058883	-0.091743	-0.000035
C	4.729808	-0.882309	-1.178397
C	4.197326	-2.160463	0.729087
C	4.197313	-2.160638	-0.728642
N	-3.830294	0.204928	-0.000006
Te	-4.384660	-1.823329	0.000250
N	-6.193789	-0.861164	0.000381
C	-7.302648	1.309824	0.000005
H	-8.271291	0.822232	0.000186
C	-7.163077	2.674737	-0.000246
H	-8.039237	3.315302	-0.000277
C	-5.857853	3.281706	-0.000442
H	-5.790273	4.366130	-0.000620
C	-4.698372	2.538730	-0.000379
H	-3.728617	3.020994	-0.000505
C	-4.797959	1.113387	-0.000146
C	-6.133096	0.465822	0.000054

Cartesian coordinates of the complex S-1 optimized at the B3LYP-D3/6-31G(d) theory level:

C	-0.010499	-0.082626	3.463156

C	0.844169	0.999727	3.013225
C	1.958164	0.417639	2.285222
C	0.579550	-1.338604	3.017466
C	1.808010	-1.043462	2.312645
C	-1.395900	0.081510	3.474173
C	-2.261635	-1.000927	3.036239
C	-3.372905	-0.414656	2.305357
C	-1.981311	1.338395	3.026443
C	-3.204299	1.032072	2.305335
C	0.283188	2.208477	2.592981
C	-1.160174	2.383046	2.600789
C	-1.524072	3.159834	1.424758
C	0.811648	2.876675	1.423937
C	-0.304527	3.467536	0.695718
C	2.492299	1.079505	1.194060
C	1.874885	2.296114	0.727231
C	1.874900	2.295946	-0.727719
C	3.076141	0.317150	-0.000003
C	2.492323	1.079229	-1.194254
C	-0.257461	-2.385078	2.607410
C	0.097348	-3.150081	1.428571
C	-1.122276	-3.461277	0.698120

C	-1.697872	-2.202839	2.602674
C	-2.235870	-2.873296	1.426039
C	2.129863	-1.757624	1.158521
C	2.778647	-1.168953	0.000167
C	2.129888	-1.757893	-1.158065
C	1.262011	-2.842335	0.723252
C	1.262027	-2.842503	-0.722562
C	-1.395829	0.080705	-3.474215
C	-1.981249	1.337694	-3.026787
C	-3.204252	1.031538	-2.305633
C	-2.261573	-1.001630	-3.036047
C	-3.372858	-0.415190	-2.305324
C	-0.010429	-0.083428	-3.463131
C	0.579612	-1.339303	-3.017138
C	1.808058	-1.043998	-2.312360
C	0.844230	0.999029	-3.013433
C	1.958211	0.417111	-2.285273
C	-1.160121	2.382444	-2.601358
C	0.283241	2.207877	-2.593481
C	0.811676	2.876346	-1.424581
C	-1.524044	3.159504	-1.425515
C	-0.304513	3.467375	-0.696521

C	-3.552510	1.775456	-1.176831
C	-2.695851	2.861584	-0.727941
C	-2.695866	2.861752	0.727230
C	-4.086305	1.107629	-0.000167
C	-3.552534	1.775729	1.176354
C	-1.697819	-2.203442	-2.602192
C	-2.235841	-2.873626	-1.425413
C	-1.122262	-3.461438	-0.697335
C	-0.257407	-2.385682	-2.606857
C	0.097377	-3.150412	-1.427833
C	-3.886394	-1.055523	-1.177177
C	-4.243532	-0.279043	-0.000008
C	-3.886418	-1.055250	1.177348
C	-3.303718	-2.310250	-0.727803
C	-3.303733	-2.310082	0.728277
N	4.601049	0.395356	0.000004
N	6.883709	-0.398986	0.000058
C	5.447859	1.399913	-0.000044
C	6.784664	0.913170	-0.000009
H	7.671400	1.538939	-0.000044
H	5.098014	2.424291	-0.000103
S	5.387532	-1.145086	0.000100

Cartesian coordinates of the complex S-2 optimized at the B3LYP-D3/6-31G(d) theory level:

C	-0.465923	-0.176030	3.463134
C	0.560568	0.744517	3.012684
C	1.560744	-0.018855	2.285482
C	-0.098166	-1.514171	3.017565
C	1.163378	-1.432715	2.314602
C	-1.803371	0.221721	3.474496
C	-2.841027	-0.697318	3.036857
C	-3.835912	0.069789	2.305584
C	-2.166250	1.559946	3.026939
C	-3.423566	1.466666	2.305776
C	0.213408	2.031423	2.594005
C	-1.179096	2.449585	2.601799
C	-1.405446	3.276961	1.425576
C	0.847209	2.598338	1.424038
C	-0.151476	3.371333	0.696187
C	2.202167	0.541151	1.194553
C	1.794351	1.842922	0.727682
C	1.794297	1.842960	-0.727727
C	2.657863	-0.309146	-0.000111

C	2.202080	0.541214	-1.194695
C	-1.101852	-2.402858	2.607650
C	-0.882950	-3.216759	1.428351
C	-2.137695	-3.315490	0.697766
C	-2.489846	-1.977439	2.602584
C	-3.134450	-2.546134	1.425681
C	1.356134	-2.188511	1.157444
C	2.089950	-1.716068	-0.000125
C	1.356050	-2.188451	-1.157666
C	0.316493	-3.111124	0.722546
C	0.316440	-3.111086	-0.722739
C	-1.803627	0.221903	-3.474359
C	-2.166473	1.560104	-3.026706
C	-3.423735	1.466786	-2.305455
C	-2.841250	-0.697159	-3.036692
C	-3.836082	0.069909	-2.305305
C	-0.466178	-0.175849	-3.463116
C	-0.098388	-1.514013	-3.017644
C	1.163208	-1.432594	-2.314770
C	0.560346	0.744675	-3.012694
C	1.560576	-0.018736	-2.285606
C	-1.179288	2.449721	-2.601592

C	0.213217	2.031558	-2.593923
C	0.847104	2.598413	-1.423972
C	-1.405552	3.277035	-1.425310
C	-0.151528	3.371370	-0.696007
C	-3.640251	2.259069	-1.176472
C	-2.610829	3.183022	-0.727481
C	-2.610775	3.182983	0.727831
C	-4.280104	1.692061	0.000198
C	-3.640164	2.259007	1.176850
C	-2.490037	-1.977303	-2.602512
C	-3.134555	-2.546060	-1.425591
C	-2.137747	-3.315454	-0.697789
C	-1.102043	-2.402722	-2.607702
C	-0.883055	-3.216685	-1.428462
C	-4.451193	-0.473540	-1.177091
C	-4.670589	0.352175	0.000177
C	-4.451107	-0.473601	1.177386
C	-4.091121	-1.809458	-0.727975
C	-4.091068	-1.809496	0.728174
N	4.164414	-0.513300	-0.000171
N	6.246503	-1.776651	-0.000305
C	7.695951	0.209819	-0.000235

H	8.604873	-0.380855	-0.000299
C	7.702096	1.578644	-0.000146
H	8.644684	2.116364	-0.000139
C	6.483348	2.330677	-0.000058
H	6.544279	3.414664	0.000011
C	5.245848	1.735455	-0.000055
H	4.330709	2.312057	0.000012
C	5.212750	0.318531	-0.000148
C	6.435691	-0.456680	-0.000237
S	4.645876	-2.168263	-0.000302

Cartesian coordinates of the complex Se-1 optimized at the B3LYP-D3/6-31G(d) theory level:

C	0.223567	-0.015762	-3.459082
C	-0.531288	1.140444	-3.010606
C	-1.692899	0.664624	-2.282142
C	-0.474535	-1.213726	-3.013563
C	-1.666788	-0.808537	-2.300426
C	1.617591	0.022984	-3.472260
C	2.382372	-1.133476	-3.032975
C	3.543014	-0.650050	-2.304974
C	2.314891	1.221888	-3.026790

C	3.504448	0.806241	-2.305143
C	0.138051	2.294431	-2.593651
C	1.591244	2.336345	-2.601052
C	2.024568	3.077638	-1.425579
C	-0.326567	3.007716	-1.424699
C	0.837961	3.494736	-0.696503
C	-2.159676	1.369138	-1.190001
C	-1.438309	2.526899	-0.727463
C	-1.438256	2.526984	0.727284
C	-2.828378	0.673690	0.000070
C	-2.159590	1.369278	1.190011
C	0.264102	-2.331214	-2.603645
C	-0.157633	-3.063896	-1.428337
C	1.029337	-3.483092	-0.697382
C	1.715805	-2.281951	-2.601957
C	2.191136	-2.997966	-1.425616
C	-2.058478	-1.499776	-1.161974
C	-2.686893	-0.860976	0.000155
C	-2.058392	-1.499639	1.162315
C	-1.289608	-2.647511	-0.723902
C	-1.289554	-2.647425	0.724323
C	1.617844	0.023393	3.472154

C	2.315111	1.222244	3.026492
C	3.504616	0.806512	2.304808
C	2.382593	-1.133118	3.032950
C	3.543182	-0.649779	2.304807
C	0.223820	-0.015354	3.459083
C	-0.474315	-1.213371	3.013756
C	-1.666619	-0.808266	2.300655
C	-0.531069	1.140799	3.010526
C	-1.692734	0.664893	2.282203
C	1.591433	2.336652	2.600676
C	0.138240	2.294737	2.593386
C	-0.326463	3.007883	1.424384
C	2.024672	3.077806	1.425084
C	0.838011	3.494818	0.696045
C	3.919367	1.515910	1.176401
C	3.164473	2.674785	0.727413
C	3.164420	2.674699	-0.727943
C	4.389504	0.801502	-0.000199
C	3.919280	1.515771	-1.176850
C	1.715996	-2.281644	2.602116
C	2.191241	-2.997799	1.425825
C	1.029387	-3.483009	0.697733

C	0.264292	-2.330906	2.603914
C	-0.157529	-3.063729	1.428725
C	3.997254	-1.334341	1.176962
C	4.423189	-0.593311	-0.000119
C	3.997170	-1.334480	-1.177081
C	3.305565	-2.532237	0.728045
C	3.305512	-2.532323	-0.727972
N	-4.307011	0.824391	0.000112
N	-6.688414	0.029352	0.000224
C	-5.155443	1.805040	0.000108
C	-6.515636	1.316322	0.000171
H	-7.361751	2.002731	0.000185
H	-4.840864	2.842653	0.000080
Se	-5.065736	-0.930306	0.000153

Cartesian coordinates of the complex Se-2 optimized at the B3LYP-D3/6-31G(d) theory level:

C	0.633070	-0.100143	-3.459637
C	-0.314775	0.903508	-3.010508
C	-1.374780	0.228933	-2.282902
C	0.158047	-1.403104	-3.014415
C	-1.087987	-1.215968	-2.303377

C	1.998390	0.185150	-3.472611
C	2.956281	-0.817455	-3.033814
C	4.012446	-0.135965	-2.305022
C	2.471997	1.488494	-3.026516
C	3.716456	1.290344	-2.304930
C	0.139379	2.157869	-2.593829
C	1.562113	2.456863	-2.600862
C	1.857081	3.262703	-1.425010
C	-0.444223	2.776078	-1.424122
C	0.615225	3.461962	-0.695784
C	-1.960409	0.837973	-1.190194
C	-1.452299	2.103824	-0.727426
C	-1.452293	2.103619	0.728026
C	-2.501412	0.034446	0.000015
C	-1.960402	0.837641	1.190443
C	1.083712	-2.372132	-2.604739
C	0.798824	-3.167774	-1.429189
C	2.041211	-3.370116	-0.698101
C	2.503395	-2.065669	-2.602390
C	3.098430	-2.686291	-1.426097
C	-1.348443	-1.963593	-1.161618
C	-2.071427	-1.443502	-0.000200

C	-1.348442	-1.963925	1.161068
C	-0.388587	-2.958879	-0.724239
C	-0.388584	-2.959084	0.723399
C	1.998413	0.184168	3.472643
C	2.472018	1.487638	3.026914
C	3.716472	1.289692	2.305263
C	2.956302	-0.818313	3.033556
C	4.012462	-0.136617	2.304950
C	0.633093	-0.101121	3.459598
C	0.158065	-1.403956	3.014010
C	-1.087974	-1.216620	2.303034
C	-0.314754	0.902657	3.010760
C	-1.374766	0.228289	2.282972
C	1.562131	2.456128	2.601539
C	0.139397	2.157137	2.594432
C	-0.444212	2.775677	1.424904
C	1.857091	3.262301	1.425914
C	0.615231	3.461766	0.696752
C	3.998888	2.061588	1.176932
C	3.050269	3.068108	0.728128
C	3.050264	3.068313	-0.727288
C	4.588601	1.442449	0.000185

C	3.998880	2.061921	-1.176383
C	2.503411	-2.066404	2.601782
C	3.098439	-2.686694	1.425310
C	2.041215	-3.370313	0.697128
C	1.083728	-2.372868	2.604054
C	0.798832	-3.168177	1.428281
C	4.580850	-0.729446	1.176908
C	4.868778	0.075552	-0.000009
C	4.580842	-0.729113	-1.177151
C	4.112807	-2.031080	0.727736
C	4.112802	-2.030874	-0.728344
N	-3.979620	-0.091758	0.000006
Se	-4.421327	-1.931679	0.000040
N	-6.170559	-1.338129	-0.000081
C	-7.500350	0.704170	-0.000042
H	-8.426214	0.139752	-0.000077
C	-7.473208	2.069514	-0.000023
H	-8.400840	2.633061	-0.000041
C	-6.232027	2.787934	0.000009
H	-6.261209	3.873564	0.000021
C	-5.016032	2.155491	0.000018
H	-4.084488	2.707196	0.000036

C	-5.008985	0.734847	0.000008
C	-6.264108	-0.024095	-0.000027

Cartesian coordinates of the complex Te-1 optimized at the B3LYP-D3/SDD theory level:

C	0.447445	0.015579	-3.481711
C	-0.262566	1.210468	-3.029627
C	-1.450725	0.783594	-2.295859
C	-0.304738	-1.158331	-3.031559
C	-1.483602	-0.699148	-2.310016
C	1.850344	-0.005786	-3.494793
C	2.569329	-1.201984	-3.051432
C	3.758267	-0.766414	-2.319917
C	2.603731	1.169490	-3.047172
C	3.781635	0.699790	-2.319913
C	0.460347	2.341865	-2.611024
C	1.923674	2.320428	-2.618007
C	2.391298	3.047176	-1.434784
C	0.023550	3.077073	-1.433260
C	1.215404	3.516906	-0.700876
C	-1.887465	1.507648	-1.195110
C	-1.115677	2.642805	-0.732068

C	-1.115629	2.642890	0.731827
C	-2.608516	0.849327	0.000030
C	-1.887383	1.507785	1.195048
C	0.388076	-2.313437	-2.618251
C	-0.068327	-3.031813	-1.436615
C	1.107449	-3.505495	-0.701492
C	1.850749	-2.328677	-2.618771
C	2.297419	-3.069162	-1.434667
C	-1.911460	-1.378783	-1.170683
C	-2.554614	-0.716884	0.000118
C	-1.911383	-1.378648	1.170953
C	-1.189663	-2.562968	-0.729230
C	-1.189615	-2.562884	0.729589
C	1.850574	-0.005384	3.494660
C	2.603932	1.169839	3.046855
C	3.781789	0.700056	2.319573
C	2.569529	-1.201634	3.051389
C	3.758420	-0.766148	2.319746
C	0.447674	0.015979	3.481669
C	-0.304538	-1.157983	3.031700
C	-1.483450	-0.698882	2.310181
C	-0.262367	1.210816	3.029495

C	-1.450573	0.783858	2.295852
C	1.923847	2.320729	2.617604
C	0.460519	2.342166	2.610715
C	0.023645	3.077238	1.432894
C	2.391393	3.047340	1.434266
C	1.215450	3.516987	0.700382
C	4.229627	1.395357	1.184301
C	3.520010	2.593253	0.732305
C	3.519962	2.593169	-0.732845
C	4.670826	0.656168	-0.000197
C	4.229549	1.395221	-1.184750
C	1.850921	-2.328376	2.618904
C	2.297513	-3.068997	1.434856
C	1.107495	-3.505414	0.701809
C	0.388248	-2.313136	2.618478
C	-0.068233	-3.031648	1.436955
C	4.185186	-1.474066	1.184678
C	4.646393	-0.747478	-0.000116
C	4.185108	-1.474202	-1.184797
C	3.438410	-2.649243	0.732833
C	3.438362	-2.649327	-0.732768
N	-4.076238	1.090781	0.000054

N	-6.604341	0.520816	0.000418
C	-4.843822	2.146187	-0.000027
C	-6.272190	1.793928	0.000339
H	-7.030102	2.575129	0.000380
H	-4.466922	3.163865	-0.000113
Te	-5.007660	-0.827940	0.000201

Cartesian coordinates of the complex Te-2 optimized at the B3LYP-D3/SDD theory level:

C	0.810190	-0.059152	-3.481587
C	-0.096287	0.994196	-3.029119
C	-1.193954	0.368466	-2.295995
C	0.272673	-1.345766	-3.031849
C	-0.968497	-1.097656	-2.311397
C	2.195802	0.162931	-3.494787
C	3.111400	-0.890419	-3.051379
C	4.206931	-0.255358	-2.319911
C	2.734275	1.450886	-3.047390
C	3.975805	1.192614	-2.320015
C	0.420033	2.233658	-2.611375
C	1.864910	2.466375	-2.618280
C	2.199664	3.262989	-1.434949

C	-0.137189	2.880551	-1.432838
C	0.960228	3.521447	-0.700989
C	-1.750967	1.004879	-1.195120
C	-1.182669	2.254364	-0.732367
C	-1.182652	2.254540	0.731854
C	-2.354165	0.231588	-0.000001
C	-1.750941	1.005167	1.194920
C	1.155806	-2.363241	-2.618240
C	0.831290	-3.149744	-1.436566
C	2.071327	-3.412715	-0.701337
C	2.598861	-2.124396	-2.618472
C	3.167496	-2.776423	-1.434559
C	-1.269280	-1.839672	-1.169815
C	-2.012433	-1.297444	0.000180
C	-1.269252	-1.839390	1.170289
C	-0.353962	-2.882214	-0.728908
C	-0.353946	-2.882039	0.729613
C	2.195882	0.163773	3.494701
C	2.734345	1.451619	3.046981
C	3.975858	1.193173	2.319641
C	3.111470	-0.889685	3.051526
C	4.206984	-0.254800	2.319880

C	0.810270	-0.058314	3.481586
C	0.272741	-1.345036	3.032171
C	-0.968444	-1.097099	2.311686
C	-0.096218	0.994926	3.028884
C	-1.193901	0.369019	2.295936
C	1.864970	2.467005	2.617647
C	0.420093	2.234286	2.610831
C	-0.137156	2.880895	1.432150
C	2.199697	3.263334	1.434116
C	0.960244	3.521615	0.700123
C	4.296456	1.955391	1.184249
C	3.390119	3.012286	0.732208
C	3.390103	3.012111	-0.733008
C	4.859323	1.303951	-0.000211
C	4.296429	1.955106	-1.184815
C	2.598921	-2.123766	2.618928
C	3.167529	-2.776079	1.435160
C	2.071343	-3.412546	0.702115
C	1.155865	-2.362610	2.618786
C	0.831323	-3.149399	1.437309
C	4.750184	-0.878124	1.184845
C	5.078843	-0.082751	-0.000046

C	4.750157	-0.878409	-1.184739
C	4.218311	-2.164899	0.733002
C	4.218294	-2.165075	-0.732573
N	-3.837895	0.195724	0.000009
Te	-4.409541	-1.834124	0.000273
N	-6.219791	-0.849298	0.000308
C	-7.306022	1.346991	-0.000044
H	-8.283119	0.875567	0.000094
C	-7.144086	2.714378	-0.000277
H	-8.010871	3.368681	-0.000333
C	-5.825496	3.305872	-0.000434
H	-5.743446	4.389826	-0.000605
C	-4.673419	2.543672	-0.000353
H	-3.695862	3.008660	-0.000457
C	-4.796285	1.116225	-0.000127
C	-6.146352	0.481728	0.000033

Cartesian coordinates of the complex S-1 optimized at the B3LYP/6-31G(d) theory level:

C	-0.012605	-0.080657	3.460503
C	0.838152	1.002636	3.010798

C	1.952740	0.424124	2.284093
C	0.580202	-1.333554	3.015160
C	1.806639	-1.035790	2.311623
C	-1.399122	0.079648	3.471950
C	-2.261043	-1.004023	3.034811
C	-3.372591	-0.421299	2.304636
C	-1.987346	1.333575	3.024796
C	-3.208298	1.024360	2.304662
C	0.273512	2.210173	2.590710
C	-1.168617	2.380876	2.598856
C	-1.534208	3.155734	1.424191
C	0.799658	2.878857	1.423151
C	-0.316887	3.466170	0.695955
C	2.488897	1.088130	1.192912
C	1.865609	2.301961	0.726670
C	1.865626	2.301795	-0.727155
C	3.078026	0.327084	-0.000002
C	2.488924	1.087856	-1.193104
C	-0.254344	-2.382863	2.605510
C	0.102080	-3.145382	1.427824
C	-1.115171	-3.460036	0.698397
C	-1.693663	-2.204854	2.600844

C	-2.229272	-2.876063	1.425570
C	2.131073	-1.749325	1.156633
C	2.781997	-1.160212	0.000164
C	2.131100	-1.749589	-1.156185
C	1.266477	-2.834185	0.722105
C	1.266494	-2.834350	-0.721430
C	-1.399040	0.078854	-3.472003
C	-1.987276	1.332884	-3.025150
C	-3.208244	1.023834	-2.304974
C	-2.260972	-1.004716	-3.034637
C	-3.372537	-0.421825	-2.304621
C	-0.012524	-0.081447	-3.460487
C	0.580272	-1.334243	-3.014843
C	1.806693	-1.036318	-2.311346
C	0.838223	1.001948	-3.011010
C	1.952793	0.423602	-2.284146
C	-1.168556	2.380282	-2.599430
C	0.273573	2.209581	-2.591211
C	0.799691	2.878531	-1.423793
C	-1.534174	3.155408	-1.424950
C	-0.316870	3.466010	-0.696757
C	-3.558630	1.767117	-1.175556

C	-2.705879	2.854403	-0.727136
C	-2.705896	2.854569	0.726418
C	-4.090106	1.098582	-0.000174
C	-3.558658	1.767386	1.175067
C	-1.693602	-2.205448	-2.600382
C	-2.229239	-2.876389	-1.424967
C	-1.115154	-3.460195	-0.697634
C	-0.254283	-2.383458	-2.604974
C	0.102113	-3.145709	-1.427104
C	-3.884684	-1.063908	-1.175978
C	-4.243425	-0.289202	-0.000018
C	-3.884712	-1.063639	1.176128
C	-3.299191	-2.315650	-0.727050
C	-3.299208	-2.315483	0.727500
N	4.617927	0.403028	0.000008
N	6.900105	-0.408550	0.000120
C	5.476353	1.399249	-0.000113
C	6.809423	0.903943	-0.000040
H	7.700495	1.524118	-0.000122
H	5.140901	2.428854	-0.000254
S	5.400656	-1.140651	0.000238

Cartesian coordinates of the complex S-2 optimized at the B3LYP/6-31G(d) theory level:

C	-0.470714	-0.170822	3.460555
C	0.547897	0.755987	3.010219
C	1.552715	0.000777	2.284312
C	-0.093618	-1.504638	3.015381
C	1.165929	-1.414311	2.313599
C	-1.811751	0.217147	3.472329
C	-2.841606	-0.708521	3.035472
C	-3.840865	0.050554	2.304862
C	-2.184052	1.551227	3.025306
C	-3.439393	1.448987	2.305134
C	0.190942	2.040635	2.591599
C	-1.202895	2.448349	2.599812
C	-1.435010	3.273019	1.425012
C	0.819967	2.611297	1.423230
C	-0.183192	3.376129	0.696465
C	2.194250	0.565500	1.193459
C	1.774572	1.863822	0.727175
C	1.774542	1.863873	-0.727098
C	2.659548	-0.281836	-0.000054
C	2.194195	0.565581	-1.193489

C	-1.091211	-2.401164	2.605824
C	-0.866509	-3.211727	1.427613
C	-2.118971	-3.320135	0.698016
C	-2.480801	-1.986577	2.600807
C	-3.120473	-2.559343	1.425187
C	1.364764	-2.168838	1.155603
C	2.099026	-1.692917	-0.000095
C	1.364711	-2.168754	-1.155793
C	0.332723	-3.096873	0.721382
C	0.332691	-3.096822	-0.721595
C	-1.811899	0.217393	-3.472216
C	-2.184181	1.551441	-3.025083
C	-3.439491	1.449150	-2.304864
C	-2.841736	-0.708306	-3.035381
C	-3.840964	0.050717	-2.304674
C	-0.470862	-0.170577	-3.460527
C	-0.093748	-1.504425	-3.015465
C	1.165828	-1.414147	-2.313729
C	0.547769	0.756200	-3.010169
C	1.552616	0.000937	-2.284357
C	-1.203006	2.448532	-2.599567
C	0.190832	2.040818	-2.591443

C	0.819907	2.611396	-1.423059
C	-1.435071	3.273119	-1.424700
C	-0.183221	3.376178	-0.696199
C	-3.661860	2.240154	-1.175226
C	-2.640356	3.170587	-0.726675
C	-2.640325	3.170536	0.727032
C	-4.296941	1.669097	0.000161
C	-3.661809	2.240071	1.175560
C	-2.480913	-1.986393	-2.600822
C	-3.120534	-2.559242	-1.425214
C	-2.119001	-3.320086	-0.698139
C	-1.091323	-2.400979	-2.605927
C	-0.866571	-3.211626	-1.427783
C	-4.452493	-0.497541	-1.175948
C	-4.677574	0.325670	0.000122
C	-4.452443	-0.497624	1.176123
C	-4.083015	-1.829330	-0.727280
C	-4.082984	-1.829382	0.727346
N	4.182495	-0.489247	-0.000099
N	6.251782	-1.783766	-0.000155
C	7.731514	0.177205	-0.000272
H	8.629631	-0.430523	-0.000285

C	7.763236	1.545717	-0.000345
H	8.715451	2.066982	-0.000415
C	6.557100	2.317721	-0.000340
H	6.635709	3.400992	-0.000400
C	5.309264	1.743498	-0.000269
H	4.406104	2.339924	-0.000269
C	5.247294	0.325698	-0.000188
C	6.458646	-0.466826	-0.000193
S	4.648661	-2.149752	0.000001

Cartesian coordinates of the complex Se-1 optimized at the B3LYP/6-31G(d) theory level:

C	0.228171	-0.018770	-3.455768
C	-0.527732	1.134809	-3.008028
C	-1.687509	0.657820	-2.280760
C	-0.467074	-1.216350	-3.009957
C	-1.659010	-0.813938	-2.298743
C	1.622776	0.022379	-3.469850
C	2.388941	-1.131293	-3.031078
C	3.547374	-0.645874	-2.304064
C	2.317247	1.221482	-3.025646
C	3.506423	0.808805	-2.304919

C	0.139908	2.290605	-2.592122
C	1.591308	2.335357	-2.600109
C	2.022909	3.077084	-1.426427
C	-0.325493	3.002682	-1.425126
C	0.836939	3.491709	-0.698267
C	-2.159612	1.363761	-1.189567
C	-1.437530	2.521177	-0.727842
C	-1.437405	2.521911	0.725707
C	-2.833680	0.667898	-0.000016
C	-2.159407	1.364960	1.188730
C	0.274068	-2.332983	-2.600505
C	-0.145711	-3.064487	-1.426107
C	1.040526	-3.481127	-0.696068
C	1.723969	-2.281159	-2.599031
C	2.200213	-2.995045	-1.423803
C	-2.051901	-1.505746	-1.158880
C	-2.686804	-0.869070	0.000768
C	-2.051681	-1.504562	1.160950
C	-1.279069	-2.650353	-0.721500
C	-1.278938	-2.649617	0.724598
C	1.623388	0.025908	3.469719
C	2.317780	1.224558	3.024171

C	3.506829	0.811148	2.303655
C	2.389476	-1.128210	3.031983
C	3.547780	-0.643530	2.304271
C	0.228781	-0.015256	3.455924
C	-0.466541	-1.213290	3.011456
C	-1.658594	-0.811600	2.300036
C	-0.527204	1.137867	3.007144
C	-1.687109	0.660137	2.280562
C	1.591764	2.337999	2.597630
C	0.140364	2.293237	2.589943
C	-0.325245	3.004126	1.422306
C	2.023160	3.078532	1.423118
C	0.837060	3.492416	0.694747
C	3.920430	1.521109	1.174305
C	3.164347	2.677212	0.725322
C	3.164220	2.676473	-0.728424
C	4.391365	0.807820	-0.000709
C	3.920224	1.519914	-1.176365
C	1.724430	-2.278517	2.601224
C	2.200466	-2.993596	1.426638
C	1.040650	-3.480418	0.699603
C	0.274529	-2.330338	2.603005

C	-0.145457	-3.063038	1.429428
C	4.003353	-1.328148	1.176199
C	4.427303	-0.587629	-0.000003
C	4.003146	-1.329344	-1.175377
C	3.314507	-2.526147	0.728317
C	3.314378	-2.526887	-0.726155
N	-4.321905	0.821225	0.000007
N	-6.711558	0.032747	0.000442
C	-5.172464	1.802688	-0.000009
C	-6.533337	1.319761	0.000158
H	-7.378017	2.008225	0.000396
H	-4.860841	2.841717	0.000036
Se	-5.098908	-0.928526	-0.000174

Cartesian coordinates of the complex Se-2 optimized at the B3LYP/6-31G(d) theory level:

C	0.640043	-0.099368	-3.456430
C	-0.304201	0.905354	-3.007632
C	-1.365296	0.234496	-2.281536
C	0.162308	-1.399475	-3.011350
C	-1.082123	-1.209698	-2.302107
C	2.006754	0.182564	-3.470286

C	2.961276	-0.821212	-3.032208
C	4.017887	-0.142801	-2.304326
C	2.483168	1.483463	-3.025222
C	3.725916	1.282760	-2.304639
C	0.153489	2.159056	-2.591815
C	1.575228	2.454559	-2.599505
C	1.871895	3.258982	-1.425302
C	-0.427896	2.777859	-1.423851
C	0.631984	3.460795	-0.696891
C	-1.954531	0.846066	-1.189592
C	-1.439285	2.109163	-0.727326
C	-1.439271	2.109339	0.726842
C	-2.505577	0.043893	0.000018
C	-1.954510	0.846355	1.189424
C	1.086158	-2.371203	-2.602168
C	0.799717	-3.164285	-1.427635
C	2.040071	-3.369800	-0.697519
C	2.504959	-2.068686	-2.600017
C	3.097910	-2.689821	-1.424957
C	-1.346056	-1.956632	-1.158850
C	-2.073012	-1.436411	0.000192
C	-1.346036	-1.956353	1.159346

C	-0.387622	-2.952450	-0.722370
C	-0.387609	-2.952276	0.723088
C	2.006819	0.183402	3.470202
C	2.483225	1.484194	3.024814
C	3.725959	1.283317	2.304257
C	2.961333	-0.820480	3.032349
C	4.017930	-0.142245	2.304283
C	0.640107	-0.098533	3.456440
C	0.162364	-1.398747	3.011683
C	-1.082081	-1.209142	2.302417
C	-0.304144	0.906081	3.007417
C	-1.365254	0.235047	2.281504
C	1.575277	2.455187	2.598880
C	0.153538	2.159683	2.591288
C	-0.427870	2.778203	1.423186
C	1.871921	3.259326	1.424477
C	0.631997	3.460964	0.696041
C	4.010399	2.054573	1.175100
C	3.065358	3.062247	0.726513
C	3.065344	3.062072	-0.727312
C	4.597999	1.434367	-0.000217
C	4.010377	2.054289	-1.175674

C	2.505008	-2.068058	2.600467
C	3.097937	-2.689477	1.425547
C	2.040084	-3.369631	0.698293
C	1.086206	-2.370574	2.602719
C	0.799743	-3.163940	1.428382
C	4.585225	-0.737082	1.175861
C	4.874704	0.066063	-0.000055
C	4.585203	-0.737366	-1.175771
C	4.114450	-2.036235	0.727478
C	4.114437	-2.036411	-0.727065
N	-3.996385	-0.081630	0.000049
Se	-4.444498	-1.924973	0.000386
N	-6.187720	-1.342019	0.000249
C	-7.531478	0.687150	-0.000079
H	-8.451124	0.111888	0.000013
C	-7.519787	2.052667	-0.000308
H	-8.453310	2.607106	-0.000405
C	-6.284868	2.781551	-0.000429
H	-6.323067	3.867307	-0.000612
C	-5.062459	2.160528	-0.000326
H	-4.139006	2.726416	-0.000423
C	-5.035908	0.737925	-0.000084

C	-6.286802	-0.028169	0.000044

Cartesian coordinates of the complex Te-1 optimized at the B3LYP/SDD theory level:

C	0.453225	0.014721	-3.479984
C	-0.257354	1.208131	-3.027924
C	-1.445027	0.780789	-2.295420
C	-0.297495	-1.159067	-3.029808
C	-1.476535	-0.701711	-2.309546
C	1.857133	-0.005438	-3.493972
C	2.576995	-1.200415	-3.051268
C	3.764970	-0.763943	-2.320188
C	2.609211	1.169974	-3.046884
C	3.787082	0.701604	-2.320195
C	0.465086	2.340908	-2.609822
C	1.927602	2.321060	-2.617294
C	2.394381	3.047843	-1.434833
C	0.027810	3.075172	-1.433023
C	1.218654	3.515981	-0.701280
C	-1.886544	1.506638	-1.195135
C	-1.112593	2.640881	-0.731897
C	-1.112549	2.640967	0.731640

C	-2.614650	0.848085	0.000018
C	-1.886468	1.506776	1.195052
C	0.396988	-2.314406	-2.617231
C	-0.058418	-3.032458	-1.436374
C	1.117053	-3.505164	-0.701925
C	1.858831	-2.328463	-2.618102
C	2.306063	-3.068240	-1.434758
C	-1.906867	-1.382829	-1.169466
C	-2.556188	-0.722390	0.000108
C	-1.906798	-1.382693	1.169719
C	-1.181265	-2.565136	-0.728660
C	-1.181222	-2.565052	0.729007
C	1.857342	-0.005033	3.493843
C	2.609394	1.170327	3.046575
C	3.787221	0.701873	2.319870
C	2.577177	-1.200061	3.051234
C	3.765109	-0.763674	2.320034
C	0.453433	0.015125	3.479938
C	-0.297314	-1.158715	3.029942
C	-1.476397	-0.701442	2.309696
C	-0.257172	1.208484	3.027783
C	-1.444887	0.781056	2.295397

C	1.927759	2.321364	2.616893
C	0.465242	2.341212	2.609507
C	0.027896	3.075339	1.432649
C	2.394467	3.048010	1.434320
C	1.218696	3.516063	0.700782
C	4.234803	1.398098	1.183744
C	3.524481	2.594774	0.731950
C	3.524437	2.594689	-0.732478
C	4.676484	0.659652	-0.000187
C	4.234731	1.397961	-1.184176
C	1.858987	-2.328159	2.618242
C	2.306148	-3.068074	1.434957
C	1.117094	-3.505082	0.702245
C	0.397144	-2.314101	2.617456
C	-0.058333	-3.032291	1.436710
C	4.192923	-1.471760	1.184150
C	4.653200	-0.745062	-0.000105
C	4.192853	-1.471897	-1.184249
C	3.447593	-2.647046	0.732499
C	3.447549	-2.647131	-0.732417
N	-4.089596	1.090350	0.000032
N	-6.626409	0.525253	0.000495

C	-4.860200	2.145851	-0.000072
C	-6.289629	1.797328	0.000446
H	-7.045680	2.580833	0.000499
H	-4.487452	3.165620	-0.000161
Te	-5.038286	-0.827587	0.000184

Cartesian coordinates of the complex Te-2 optimized at the B3LYP/SDD theory level:

C	0.818111	-0.057590	-3.479887
C	-0.085654	0.996769	-3.027232
C	-1.184795	0.374009	-2.295577
C	0.278290	-1.342248	-3.030286
C	-0.961960	-1.092168	-2.311050
C	2.205176	0.161773	-3.493997
C	3.118340	-0.892913	-3.051242
C	4.214647	-0.260360	-2.320205
C	2.746150	1.448032	-3.047129
C	3.986648	1.187430	-2.320320
C	0.433710	2.235876	-2.610027
C	1.878176	2.465869	-2.617543
C	2.214399	3.261335	-1.434982
C	-0.121892	2.883149	-1.432455

C	0.976139	3.521648	-0.701360
C	-1.745723	1.012907	-1.195201
C	-1.170728	2.259927	-0.732145
C	-1.170712	2.260103	0.731627
C	-2.359648	0.240540	-0.000004
C	-1.745699	1.013195	1.194995
C	1.160105	-2.362216	-2.617295
C	0.834213	-3.147155	-1.436343
C	2.072965	-3.412997	-0.701777
C	2.602842	-2.126658	-2.617859
C	3.169962	-2.779562	-1.434675
C	-1.266663	-1.834664	-1.168520
C	-2.014674	-1.292723	0.000178
C	-1.266637	-1.834382	1.168989
C	-0.351320	-2.877483	-0.728294
C	-0.351305	-2.877309	0.728996
C	2.205252	0.162613	3.493913
C	2.746215	1.448765	3.046723
C	3.986698	1.187988	2.319950
C	3.118406	-0.892180	3.051392
C	4.214696	-0.259801	2.320178
C	0.818186	-0.056753	3.479885

C	0.278355	-1.341520	3.030606
C	-0.961910	-1.091612	2.311335
C	-0.085589	0.997497	3.026995
C	-1.184746	0.374562	2.295514
C	1.878232	2.466499	2.616910
C	0.433766	2.236504	2.609481
C	-0.121860	2.883492	1.431765
C	2.214430	3.261679	1.434150
C	0.976154	3.521816	0.700493
C	4.309109	1.950130	1.183694
C	3.405367	3.008326	0.731855
C	3.405352	3.008151	-0.732651
C	4.870394	1.297838	-0.000208
C	4.309084	1.949845	-1.184255
C	2.602899	-2.126028	2.618317
C	3.169993	-2.779218	1.435278
C	2.072980	-3.412829	0.702556
C	1.160161	-2.361586	2.617841
C	0.834244	-3.146810	1.437085
C	4.757102	-0.884710	1.184317
C	5.087172	-0.090352	-0.000043
C	4.757077	-0.884995	-1.184205

C	4.222873	-2.169772	0.732660
C	4.222857	-2.169948	-0.732227
N	-3.853008	0.204928	0.000003
Te	-4.431728	-1.833023	0.000268
N	-6.238174	-0.853846	0.000337
C	-7.338307	1.332689	-0.000026
H	-8.310864	0.851009	0.000130
C	-7.190609	2.701725	-0.000268
H	-8.063689	3.348208	-0.000314
C	-5.876598	3.303755	-0.000440
H	-5.803006	4.388747	-0.000615
C	-4.717150	2.551527	-0.000366
H	-3.745549	3.030282	-0.000478
C	-4.821385	1.120051	-0.000136
C	-6.169492	0.477433	0.000040

Cartesian coordinates of the complex S-1 optimized at the M06-2X/6-31G(d) theory level:

C	-0.018455	-0.096807	3.451796
C	0.853223	0.966930	3.002164

C	1.953417	0.364046	2.277470
C	0.548341	-1.359909	3.005883
C	1.781554	-1.090320	2.310547
C	-1.393412	0.091690	3.461987
C	-2.277256	-0.971719	3.026201
C	-3.374495	-0.365903	2.296315
C	-1.953934	1.356344	3.015534
C	-3.180009	1.073610	2.296491
C	0.318211	2.179638	2.584358
C	-1.118578	2.380310	2.592176
C	-1.467362	3.161996	1.419743
C	0.857504	2.836841	1.419174
C	-0.245512	3.446847	0.692769
C	2.498295	1.012608	1.190221
C	1.904372	2.240442	0.726740
C	1.904364	2.240622	-0.726210
C	3.062916	0.241342	0.000014
C	2.498276	1.012899	-1.189999
C	-0.304259	-2.386081	2.600099
C	0.035682	-3.151198	1.422488
C	-1.186320	-3.440218	0.693496
C	-1.737321	-2.176015	2.593471

C	-2.286817	-2.834699	1.419836
C	2.084321	-1.798574	1.152167
C	2.732155	-1.220168	-0.000174
C	2.084297	-1.798281	-1.152649
C	1.199999	-2.868059	0.719870
C	1.199988	-2.867878	-0.720612
C	-1.393457	0.092556	-3.461956
C	-1.953973	1.357099	-3.015181
C	-3.180040	1.074186	-2.296192
C	-2.277295	-0.970962	-3.026425
C	-3.374525	-0.365328	-2.296376
C	-0.018500	-0.095944	-3.451829
C	0.548299	-1.359158	-3.006239
C	1.781518	-1.089741	-2.310850
C	0.853187	0.967679	-3.001941
C	1.953388	0.364613	-2.277406
C	-1.118609	2.380957	-2.591577
C	0.318182	2.180283	-2.583829
C	0.857489	2.837195	-1.418485
C	-1.467378	3.162350	-1.418944
C	-0.245519	3.447019	-0.691915
C	-3.513028	1.820269	-1.173433

C	-2.638657	2.887860	-0.725231
C	-2.638648	2.887677	0.725976
C	-4.058276	1.164080	0.000166
C	-3.513012	1.819974	1.173922
C	-1.737355	-2.175366	-2.593999
C	-2.286835	-2.834341	-1.420523
C	-1.186329	-3.440044	-0.694349
C	-0.304294	-2.385433	-2.600700
C	0.035663	-3.150843	-1.423285
C	-3.897668	-0.992913	-1.174681
C	-4.239325	-0.212120	-0.000005
C	-3.897651	-0.993208	1.174471
C	-3.338788	-2.254929	-0.726561
C	-3.338778	-2.255111	0.726030
N	4.613165	0.335834	0.000010
N	6.907771	-0.360041	0.000059
C	5.413950	1.373329	-0.000047
C	6.768536	0.944699	-0.000037
H	7.635121	1.595150	-0.000017
H	5.011570	2.379593	-0.000069
S	5.462128	-1.125350	-0.000002

Cartesian coordinates of the complex S-2 optimized at the M06-2X/6-31G(d) theory level:

C	-0.469498	-0.180944	3.450993
C	0.560312	0.730291	3.000486
C	1.552960	-0.038818	2.276801
C	-0.110496	-1.518036	3.005751
C	1.150135	-1.447550	2.311288
C	-1.797354	0.223438	3.461861
C	-2.839140	-0.686063	3.026560
C	-3.826209	0.086010	2.296399
C	-2.150564	1.560948	3.015608
C	-3.405869	1.476438	2.296498
C	0.223483	2.012832	2.584725
C	-1.163246	2.439450	2.592638
C	-1.383659	3.266377	1.419887
C	0.858837	2.574073	1.418576
C	-0.132564	3.352090	0.692476
C	2.198184	0.514142	1.190769
C	1.796200	1.816833	0.726677
C	1.796178	1.816887	-0.726565
C	2.647093	-0.337097	-0.000040
C	2.198140	0.514228	-1.190767

C	-1.115909	-2.396227	2.600673
C	-0.902836	-3.205990	1.423179
C	-2.155013	-3.297233	0.693994
C	-2.497240	-1.960933	2.593920
C	-3.144618	-2.524049	1.420303
C	1.333094	-2.193087	1.151546
C	2.063422	-1.725030	-0.000081
C	1.333052	-2.193001	-1.151716
C	0.290996	-3.109680	0.719905
C	0.290971	-3.109627	-0.720108
C	-1.797474	0.223695	-3.461743
C	-2.150669	1.561172	-3.015378
C	-3.405949	1.476608	-2.296232
C	-2.839246	-0.685839	-3.026474
C	-3.826289	0.086180	-2.296221
C	-0.469618	-0.180688	-3.450952
C	-0.110602	-1.517814	-3.005822
C	1.150053	-1.447379	-2.311397
C	0.560208	0.730512	-3.000412
C	1.552880	-0.038651	-2.276819
C	-1.163335	2.439642	-2.592376
C	0.223394	2.013023	-2.584542

C	0.858789	2.574178	-1.418375
C	-1.383708	3.266482	-1.419556
C	-0.132587	3.352141	-0.692182
C	-3.616503	2.266279	-1.173492
C	-2.583676	3.181421	-0.725434
C	-2.583652	3.181367	0.725799
C	-4.259056	1.705205	0.000157
C	-3.616463	2.266192	1.173825
C	-2.497331	-1.960742	-2.593941
C	-3.144668	-2.523944	-1.420342
C	-2.155038	-3.297182	-0.694125
C	-1.116000	-2.396035	-2.600774
C	-0.902886	-3.205885	-1.423347
C	-4.442562	-0.450241	-1.174380
C	-4.655771	0.374947	0.000114
C	-4.442521	-0.450328	1.174540
C	-4.091365	-1.784969	-0.726284
C	-4.091339	-1.785023	0.726332
N	4.168704	-0.540175	-0.000091
N	6.248995	-1.764856	-0.000071
C	7.684961	0.229825	-0.000273
H	8.598950	-0.352586	-0.000250

C	7.671099	1.591390	-0.000378
H	8.604654	2.143407	-0.000444
C	6.439649	2.328883	-0.000401
H	6.489472	3.412948	-0.000483
C	5.214256	1.725554	-0.000316
H	4.291595	2.291916	-0.000329
C	5.202641	0.304138	-0.000203
C	6.428824	-0.447889	-0.000187
S	4.677981	-2.147614	-0.000077

Cartesian coordinates of the complex Se-1 optimized at the M06-2X/6-31G(d) theory level:

C	0.225474	-0.019756	-3.446052
C	-0.532864	1.130258	-2.998282
C	-1.689309	0.650125	-2.273512
C	-0.465653	-1.218001	-3.001011
C	-1.658396	-0.821603	-2.294699
C	1.611957	0.025487	-3.459115
C	2.381125	-1.124399	-3.022109
C	3.536589	-0.636381	-2.295626
C	2.302018	1.224904	-3.015209
C	3.491119	0.815855	-2.295794

C	0.127718	2.281831	-2.584416
C	1.577568	2.330263	-2.591801
C	2.006676	3.071916	-1.419967
C	-0.338329	2.991568	-1.419590
C	0.821466	3.482775	-0.692810
C	-2.157557	1.349241	-1.185779
C	-1.442563	2.509678	-0.726361
C	-1.442510	2.509763	0.726192
C	-2.822054	0.651880	0.000075
C	-2.157470	1.349379	1.185800
C	0.275136	-2.327034	-2.595811
C	-0.141263	-3.057473	-1.423401
C	1.044557	-3.470551	-0.693645
C	1.723276	-2.269433	-2.593161
C	2.201211	-2.981642	-1.420217
C	-2.042410	-1.506786	-1.156446
C	-2.679008	-0.874680	0.000163
C	-2.042317	-1.506646	1.156797
C	-1.269707	-2.649643	-0.721908
C	-1.269652	-2.649558	0.722338
C	1.612215	0.025897	3.459013
C	2.302243	1.225262	3.014913

C	3.491290	0.816127	2.295458
C	2.381351	-1.124041	3.022086
C	3.536760	-0.636109	2.295459
C	0.225731	-0.019348	3.446060
C	-0.465429	-1.217646	3.001213
C	-1.658222	-0.821331	2.294942
C	-0.532641	1.130613	2.998209
C	-1.689140	0.650393	2.273582
C	1.577761	2.330570	2.591428
C	0.127910	2.282137	2.584157
C	-0.338224	2.991736	1.419282
C	2.006781	3.072084	1.419474
C	0.821517	3.482857	0.692357
C	3.901072	1.523943	1.173391
C	3.142835	2.676551	0.725438
C	3.142781	2.676465	-0.725968
C	4.374002	0.813537	-0.000201
C	3.900984	1.523804	-1.173841
C	1.723471	-2.269126	2.593323
C	2.201318	-2.981474	1.420428
C	1.044610	-3.470469	0.694000
C	0.275331	-2.326726	2.596088

C	-0.141155	-3.057304	1.423796
C	3.992356	-1.315171	1.174019
C	4.413695	-0.573677	-0.000120
C	3.992268	-1.315310	-1.174140
C	3.307725	-2.513228	0.726234
C	3.307670	-2.513314	-0.726162
N	-4.304034	0.799847	0.000119
N	-6.656776	0.020602	0.000137
C	-5.138064	1.787610	0.000130
C	-6.500383	1.304017	0.000181
H	-7.349762	1.983392	0.000168
H	-4.806234	2.820268	0.000112
Se	-5.064995	-0.913847	0.000138

Cartesian coordinates of the complex Se-2 optimized at the M06-2X/6-31G(d) theory level:

C	0.634602	-0.104282	-3.446298
C	-0.314165	0.893959	-2.997892
C	-1.369460	0.217594	-2.274082
C	0.164612	-1.405444	-3.001329
C	-1.080306	-1.225195	-2.297334
C	1.991769	0.184038	-3.459412

C	2.951425	-0.812468	-3.022615
C	4.002651	-0.128484	-2.295769
C	2.460043	1.486090	-3.015351
C	3.702702	1.293021	-2.295881
C	0.133546	2.143816	-2.584793
C	1.552269	2.446576	-2.592228
C	1.844120	3.251960	-1.420292
C	-0.449908	2.759035	-1.419407
C	0.605068	3.447088	-0.692973
C	-1.956459	0.822356	-1.186520
C	-1.451349	2.088377	-0.726871
C	-1.451368	2.088575	0.726278
C	-2.494457	0.018824	-0.000030
C	-1.956484	0.822675	1.186259
C	1.089758	-2.366854	-2.596334
C	0.808738	-3.158499	-1.423393
C	2.048615	-3.356652	-0.693376
C	2.504933	-2.055001	-2.592773
C	3.101130	-2.671720	-1.419744
C	-1.334855	-1.964573	-1.155366
C	-2.063615	-1.451107	0.000184
C	-1.334872	-1.964251	1.155888

C	-0.373290	-2.955559	-0.721331
C	-0.373304	-2.955361	0.722146
C	1.991694	0.184986	3.459417
C	2.459978	1.486917	3.015010
C	3.702652	1.293651	2.295620
C	2.951361	-0.811639	3.022915
C	4.002602	-0.127853	2.295904
C	0.634527	-0.103337	3.446354
C	0.164549	-1.404621	3.001732
C	-1.080352	-1.224564	2.297660
C	-0.314231	0.894780	2.997653
C	-1.369508	0.218216	2.274002
C	1.552212	2.447286	2.591604
C	0.133488	2.144523	2.584221
C	-0.449942	2.759423	1.418653
C	1.844088	3.252350	1.419455
C	0.605051	3.447278	0.692055
C	3.981462	2.062417	1.173391
C	3.031954	3.063309	0.725345
C	3.031970	3.063110	-0.726105
C	4.572293	1.446549	-0.000142
C	3.981489	2.062096	-1.173858

C	2.504878	-2.054290	2.593404
C	3.101099	-2.671329	1.420557
C	2.048600	-3.356461	0.694353
C	1.089703	-2.366142	2.597020
C	0.808708	-3.158107	1.424289
C	4.570994	-0.715959	1.174393
C	4.855418	0.087893	0.000047
C	4.571019	-0.716282	-1.174085
C	4.108242	-2.015918	0.726556
C	4.108259	-2.016119	-0.725901
N	-3.976343	-0.110648	-0.000035
Se	-4.429738	-1.909135	-0.000147
N	-6.146460	-1.333448	-0.000008
C	-7.484321	0.705074	-0.000009
H	-8.410845	0.142056	-0.000004
C	-7.447996	2.062720	0.000015
H	-8.370275	2.633617	0.000037
C	-6.198968	2.777011	0.000022
H	-6.226132	3.862197	0.000045
C	-4.990601	2.146699	0.000010
H	-4.054853	2.693246	0.000024
C	-4.995857	0.721493	-0.000025

C	-6.246468	-0.025670	-0.000027

Cartesian coordinates of the complex Te-1 optimized at the M06-2X/SDD theory level:

C	0.447207	0.014629	-3.466626
C	-0.262256	1.205941	-3.015462
C	-1.447928	0.779909	-2.286245
C	-0.302244	-1.157113	-3.017675
C	-1.478790	-0.702199	-2.300106
C	1.841078	-0.005248	-3.480131
C	2.560071	-1.197622	-3.038422
C	3.746014	-0.761865	-2.309288
C	2.591844	1.167858	-3.034313
C	3.767668	0.700470	-2.309086
C	0.455330	2.331382	-2.600800
C	1.915431	2.310780	-2.607959
C	2.381492	3.036430	-1.428428
C	0.019731	3.064901	-1.427337
C	1.208548	3.504531	-0.696435
C	-1.881594	1.498846	-1.190213
C	-1.112641	2.634464	-0.730625
C	-1.112597	2.634550	0.730368

C	-2.604750	0.844036	0.000016
C	-1.881517	1.498982	1.190128
C	0.388211	-2.304285	-2.608102
C	-0.065767	-3.020644	-1.430793
C	1.107693	-3.492177	-0.696917
C	1.847684	-2.317948	-2.609044
C	2.294171	-3.056074	-1.428272
C	-1.904949	-1.376905	-1.166464
C	-2.559052	-0.719204	0.000107
C	-1.904884	-1.376770	1.166718
C	-1.181015	-2.556540	-0.727337
C	-1.180973	-2.556456	0.727685
C	1.841282	-0.004845	3.480006
C	2.592023	1.168209	3.034010
C	3.767804	0.700737	2.308766
C	2.560248	-1.197270	3.038393
C	3.746149	-0.761597	2.309139
C	0.447411	0.015031	3.466582
C	-0.302067	-1.156763	3.017810
C	-1.478655	-0.701931	2.300256
C	-0.262080	1.206291	3.015323
C	-1.447791	0.780174	2.286220

C	1.915585	2.311081	2.607563
C	0.455484	2.331683	2.600488
C	0.019816	3.065067	1.426966
C	2.381577	3.036595	1.427921
C	1.208590	3.504612	0.695942
C	4.212597	1.392360	1.181251
C	3.503564	2.586582	0.730369
C	3.503521	2.586498	-0.730892
C	4.653622	0.655244	-0.000183
C	4.212528	1.392223	-1.181678
C	1.847837	-2.317647	2.609186
C	2.294256	-3.055908	1.428473
C	1.107734	-3.492096	0.697238
C	0.388363	-2.303983	2.608329
C	-0.065683	-3.020477	1.431129
C	4.171423	-1.464784	1.181669
C	4.631052	-0.739367	-0.000102
C	4.171353	-1.464922	-1.181761
C	3.427770	-2.637791	0.730967
C	3.427726	-2.637876	-0.730880
N	-4.068071	1.086679	0.000022
N	-6.565862	0.515673	0.000493

C	-4.826290	2.140645	-0.000103
C	-6.259294	1.784196	0.000505
H	-7.019929	2.559418	0.000547
H	-4.445061	3.156528	-0.000197
Te	-4.987106	-0.824849	0.000173

Cartesian coordinates of the complex Te-2 optimized at the M06-2X/SDD theory level:

C	0.809425	-0.058767	-3.466110
C	-0.095205	0.991756	-3.014441
C	-1.191363	0.368213	-2.286128
C	0.273511	-1.342453	-3.017816
C	-0.964557	-1.097049	-2.301312
C	2.185953	0.162305	-3.479963
C	3.100166	-0.887986	-3.038290
C	4.193274	-0.253935	-2.309416
C	2.723099	1.447300	-3.034435
C	3.962100	1.190036	-2.309282
C	0.417863	2.224040	-2.600838
C	1.859515	2.456013	-2.607937
C	2.193679	3.251011	-1.428372
C	-0.137539	2.869786	-1.426687

C	0.957508	3.509240	-0.696471
C	-1.745536	1.000704	-1.190384
C	-1.177569	2.249252	-0.730888
C	-1.177566	2.249422	0.730336
C	-2.352114	0.230874	-0.000042
C	-1.745530	1.000980	1.190121
C	1.152275	-2.353008	-2.607848
C	0.829300	-3.137048	-1.430625
C	2.066597	-3.399233	-0.696738
C	2.592039	-2.114303	-2.608550
C	3.159862	-2.764389	-1.428040
C	-1.264666	-1.833847	-1.165601
C	-2.017610	-1.297620	0.000139
C	-1.264666	-1.833574	1.166007
C	-0.348880	-2.872147	-0.727001
C	-0.348881	-2.871979	0.727654
C	2.185952	0.163122	3.479906
C	2.723097	1.448012	3.034075
C	3.962100	1.190577	2.308984
C	3.100166	-0.887274	3.038480
C	4.193273	-0.253394	2.309456
C	0.809424	-0.057954	3.466104

C	0.273509	-1.341746	3.018112
C	-0.964558	-1.096510	2.301548
C	-0.095205	0.992463	3.014187
C	-1.191361	0.368748	2.286017
C	1.859515	2.456625	2.607340
C	0.417863	2.224649	2.600294
C	-0.137537	2.870117	1.425990
C	2.193679	3.251344	1.427587
C	0.957508	3.509403	0.695625
C	4.281086	1.948319	1.181325
C	3.376786	3.002408	0.730291
C	3.376787	3.002238	-0.731015
C	4.842886	1.298563	-0.000162
C	4.281086	1.948042	-1.181801
C	2.592038	-2.113691	2.609029
C	3.159862	-2.764056	1.428671
C	2.066597	-3.399071	0.697518
C	1.152274	-2.352396	2.608381
C	0.829299	-3.136714	1.431342
C	4.733914	-0.872577	1.181998
C	5.061799	-0.078968	0.000000
C	4.733916	-0.872853	-1.181812

C	4.204031	-2.156303	0.731168
C	4.204031	-2.156473	-0.730680
N	-3.831839	0.192236	-0.000054
Te	-4.389583	-1.825560	0.000180
N	-6.184457	-0.848787	0.000705
C	-7.292673	1.335935	0.000203
H	-8.265883	0.859184	0.000561
C	-7.133497	2.694303	-0.000171
H	-7.998148	3.348253	-0.000132
C	-5.813132	3.289640	-0.000553
H	-5.736969	4.372355	-0.000798
C	-4.664498	2.538394	-0.000546
H	-3.686662	3.004750	-0.000777
C	-4.785448	1.107767	-0.000230
C	-6.129045	0.472453	0.000166
